# The addition of bortezomib to rituximab, high-dose cytarabine and dexamethasone in relapsed or refractory mantle cell lymphoma—*a randomized, open-label phase III trial of the European mantle cell lymphoma network*

**DOI:** 10.1038/s41375-024-02254-2

**Published:** 2024-04-27

**Authors:** Luca Fischer, Linmiao Jiang, Jan Dürig, Christian Schmidt, Stephan Stilgenbauer, Krimo Bouabdallah, Philippe Solal-Celigny, Christian W. Scholz, Pierre Feugier, Maike de Wit, Ralf Ulrich Trappe, Michael Hallek, Ullrich Graeven, Mathias Hänel, Martin Hoffmann, Vincent Delwail, Margaret Macro, Jochen Greiner, Aristoteles A. N. Giagounidis, Beate Dargel, Eric Durot, Charles Foussard, Elisabeth Silkenstedt, Oliver Weigert, Christiane Pott, Wolfram Klapper, Wolfgang Hiddemann, Michael Unterhalt, Eva Hoster, Vincent Ribrag, Martin Dreyling

**Affiliations:** 1 Department of Medicine III, LMU University Hospital, LMU Munich, Munich, Germany; 2 Institute for Medical Information Processing, Biometry and Epidemiology, LMU Munich, Munich, Germany; 3 Internal Medicine, St. Josef Hospital, Essen-Werden, Germany; 4 Klinik für Innere Medizin III, Ulm University-Medical Center, Ulm, Germany; 5 Department of Hematology and Cellular Therapy, University Hospital of Bordeaux, F-33000 Bordeaux, France; 6 Institut de Cancérologie de l’Ouest, Saint-Herblain, Bld Jacques Monod, 44805 Saint-Herblain, Cedex France; 7 Department of Hematology and Oncology, Vivantes Klinikum Am Urban, Berlin, Germany; 8 Service d’Hématologie et Medecine Interne, Centre Hospitalier Universitaire Nancy-Brabois, Vandoeuvre, France; 9 Klinik für Innere Medizin, Hämatologie, Onkologie und Palliativmedizin, Vivantes Klinikum Neukölln, Berlin, Germany; 10 Department of Hematology and Oncology, DIAKO Ev. Diakonie-Krankenhaus, Bremen, Germany; 11 Department I for Internal Medicine and Centre of Integrated Oncology Aachen, Bonn, Cologne, Duesseldorf, Faculty of Medicine and University Hospital Cologne, University of Cologne, Cologne, Germany; 12 Department for Hematology, Oncology and Gastroenterology, Kliniken Maria-Hilf Mönchengladbach, Mönchengladbach, Germany; 13 Department of Internal Medicine III, Klinikum Chemnitz, Chemnitz, Germany; 14 Medizinische Klinik A, Klinikum der Stadt Ludwigshafen gGmbH, Ludwigshafen, Germany; 15 CHU Poitiers, Poitiers, France; 16 Hematology Department, University Hospital, Caen, France; 17 Department. of Internal Medicine, Diakonie-Hospital Stuttgart, 70176 Stuttgart, Germany; 18 Marien Hospital Düsseldorf, Düsseldorf, 40479 Germany; 19 Medical Center Harz GmbH, Wernigerode, Germany; 20 CHU Reims, Hématologie Clinique, F-51100 Reims, France; 21 Department of Pathology, Centre Hospitalier, 49100 Angers, France; 22 Department of Internal Medicine II: Hematology and Oncology, University Medical Center Schleswig-Holstein, Campus Kiel, Kiel, Germany; 23 Hematopathology Section, Christian-Albrechts University, Kiel, Germany; 24 Gustave Roussy, Université Paris-Saclay, DITEP, INSERM U1170 Villejuif, France

**Keywords:** B-cell lymphoma, Targeted therapies

## Abstract

The therapy of relapsed or refractory (r/r) mantle cell lymphoma (MCL) patients remains a major clinical challenge to date. We conducted a randomized, open-label, parallel-group phase-III trial hypothesizing superior efficacy of rituximab, high-dose cytarabine and dexamethasone with bortezomib (R-HAD + B) versus without (R-HAD) in r/r MCL ineligible for or relapsed after autologous stem cell transplant (ASCT). Primary endpoint was time to treatment failure (TTF), secondary endpoints included response rates, progression free survival, overall survival, and safety. In total, 128 of 175 planned patients were randomized to R-HAD + B (*n* = 64) or R-HAD (*n* = 64). Median TTF was 12 vs. 2.6 months (*p* = 0.045, MIPI-adjusted HR 0.69; 95%CI 0.47–1.02). Overall and complete response rates were 63 vs. 45% (*p* = 0.049) and 42 vs. 19% (*p* = 0.0062). A significant treatment effect was seen in the subgroup of patients >65 years (aHR 0.48, 0.29–0.79) and without previous ASCT (aHR 0.52, 0.28–0.96). Toxicity was mostly hematological and attributable to the chemotherapeutic backbone. Grade ≥3 leukocytopenia and lymphocytopenia were more common in R-HAD + B without differences in severe infections between both arms. Bortezomib in combination with chemotherapy can be effective in r/r MCL and should be evaluated further as a therapeutic option, especially if therapy with BTK inhibitors is not an option. Trial registration: NCT01449344.

## Introduction

Mantle Cell Lymphoma (MCL) is a rare B-cell neoplasia with an incidence of 1–2 per 100,000 persons per year and a male predominance of 3:1. Incidence increases with age and median age at diagnosis is between 60–70 years. The majority of MCL is derived from antigen naïve B-cells and >90% of patients carry the hallmark translocation t(11;14)(q13;q32) resulting in cyclin D1 overexpression.

The clinical course of MCL can be highly variable, ranging from indolent to aggressive with 5-year overall survival probabilities ranging from <20% to >80% depending on the risk profile [1, 2]. Several clinical and biological factors associated with a more aggressive course of disease have been identified. The most widely validated biological factors are a Ki-67 proliferation index ≥30%, blastoid morphologic variant and p53 overexpression or TP53 mutations [1–3]. The most widely validated clinical prognostic score is the MCL international prognostic index (MIPI) first published in 2008, including age, ECOG performance status, leukocyte count and LDH levels [4].

Even though prognosis has improved drastically over the last two decades, largely through the implementation of intensive, high-dose cytarabine containing first line regimens and rituximab maintenance therapy [5–7], MCL is still considered incurable and survival in relapsed or refractory (r/r) patients is short [8].

The current standard of care in first line was defined by the phase III MCL younger trial, which tested an alternating regime consisting of cyclophosphamide, doxorubicin, vincristine and prednisolone (CHOP) and dexamethasone, high-dose cytarabine and cisplatin (DHAP) plus rituximab followed by consolidating high-dose chemotherapy with autologous stem cell transplantation (ASCT) versus six cycles of R-CHOP + ASCT. The intensification increased the median FFS from 3.9 to 8.4 years and the 10-year OS from 55 to 60% in young and fit patients [7]. Treatment options for elderly or unfit patients are limited to less toxic regimens like bendamustine + rituximab (BR) or bortezomib, rituximab, cyclophosphamide, doxorubicine and prednisolone (VR-CAP). Recently, results of large first line trials have shown promising results after adding BTK inhibitors (BTKi) to standard first line regimens [9–11].

At relapse, a wide variety of therapeutic agents are available now, including conventional chemotherapeutic regimes like rituximab, bendamustine, cytarabine (R-BAC) and targeted therapies like BTKi as well as CAR-T cell therapies in later relapses. However, remissions are mostly short, with median PFS and OS times ranging from 7–25 months and 2–3 years after first relapse, with much shorter survival times in early relapsing patients [8].

During the time when we designed our trial in 2011, the only targeted agent in Europe for relapsed patients not qualifying for ASCT was temsirolimus, with a median PFS of 4.8 months (in heavily pretreated patients) [12]. Other options included chemo-immunotherapies like R-FCM (fludarabine, cyclophosphamide, mitoxantrone) with a median PFS of 8 months [13]. High-dose cytarabine had proven effective in several phase II clinical trials in first line therapy of fit MCL patients and the MCL younger trial had been initiated. Bortezomib had been tested in a small number of relapsed MCL patients showing promising results and single agent efficacy (ORR 33–45%) [14]. Furthermore, preclinical data and a small case series suggested synergistic effects when bortezomib was combined with cytarabine [15, 16]. To improve the dismal outcome in r/r MCL patients, we designed a randomized phase III trial for patients either unfit for or who had prior high-dose chemotherapy with autologous stem cell transplantation, testing the addition of the first-in class proteasome inhibitor bortezomib to rituximab and a chemotherapy backbone consisting of high-dose cytarabine (HD-cytarabine) and dexamethasone (R-HAD). Our study objective was to test for superior efficacy and compare safety when adding bortezomib to R-HAD (R-HAD + B) to R-HAD alone in r/r MCL patients.

## Material / Methods

### Study design and patients

This was a randomized, parallel-group, multicenter, international, open-label phase III clinical trial. Adult patients with a confirmed histopathological diagnosis of MCL according to WHO classification, ECOG performance state 0–2, adequate organ function and 1–3 prior lines of lymphoma therapy were included. Furthermore, patients were required to be either ineligible for or have had previous ASCT. Pretreatment with rituximab or HD-cytarabine was allowed, if the relapse occurred ≥ 12 weeks or ≥ 6 months after the last dose, respectively. Patients with sensory polyneuropathy CTCAE grade >2, symptomatic degenerative or toxic encephalopathy, an active systemic infection, HIV or hepatitis B and C as well as pregnant or breast-feeding female patients were excluded. Additionally, patients treated with anti-neoplastic therapy within 4 weeks, radioimmunoconjugates or toxin immunoconjugates within 12 weeks or within another clinical trial within 30 days before planned day 1 of cycle 1 were excluded. A comprehensive list of in- and exclusion criteria can be found in the trial protocol (supplemental information). Reference pathology review was planned for all included patients.

Patients were randomized 1:1 to R-HAD + B or R-HAD. The randomization was done centrally at the data center in Munich, was computer-controlled, stratified with permuted blocks. The selection of blocks was done via the randomization function of the Electronic Case Report Form (ECRF). The stratification was carried out based on the following parameters: Response to initial therapy (relapse vs. primary refractory disease), International Prognostic Index (IPI; 0–2 vs. 3–5), previous ASCT (yes vs. no), previous therapy with HD-cytarabine (yes vs. no) and study group (LYSA, France vs. GLSG, Germany).

All patients provided written informed consent. The trial was performed in accordance with local regulations and approved by the responsible ethics committees (Ethikkomission der medizinischen Fakultät der LMU München, CPP Ile-de-France VII). The trial was preregistered with Eudra-CT-No.: 2005-005144-62 and ClinicalTrials.gov No. NCT01449344.

### Treatment protocol

R-HAD was given in both treatment arms in 3-week intervals for a total of 4 planned cycles: Rituximab 375 mg/m² IV, d1; cytarabine 2000 mg/m² (patients >65 years; prior ASCT: 1000 mg/m²) IV, d 2 and 3; Dexamethasone 40 mg PO d 1- 4. Bortezomib 1.5 mg/m² SC, was additionally given for 4 cycles in the experimental arm on day 1 and 4. Initially, no maintenance therapy was planned, but became optional by a later study amendment. However, due to the late implementation, no patient received maintenance therapy inside the trial.

### Outcome

The primary trial endpoint was time to treatment failure (TTF). Secondary endpoints were complete response (CR) rate, overall response (OR) rate, progression-free survival (PFS), duration of response (DOR), time to next lymphoma treatment (TTNLT), overall survival (OS), safety and tolerability.

TTF was defined as the time from randomization to progressive disease (PD) or stable disease (SD) following induction therapy, or relapse or progression after complete or partial remission (CR, CRu, PR), or death from any cause, whichever occurred first. CR and OR (CR, CRu, PR) rates were assessed after induction therapy according to the International Workshop to Standardize Response Criteria for Non-Hodgkin’s Lymphoma (supplemental methods). CR rates including/excluding CRu were evaluated separately. PFS was defined as time from randomization to first documentation of PD, relapse, or death from any cause, whichever occurred first. DOR was the time from the end of successful (CR, CRu, PR) trial therapy to first documentation of progression, relapse or death from any cause, whichever occurred first. Patients with no event during follow-up were censored at the day of the last follow-up staging for FFS, PFS, and DOR. TTNLT was the time from treatment start to the start of the next lymphoma treatment outside the protocol. Patients in which no further treatment was started were censored at the day of the last follow-up staging. OS was the time from randomization to death. Patients who were alive at the day of the last contact were censored at that time. For the per-protocol analysis, patients with new lymphoma treatment before progression were censored at treatment start.

The treatment outcome was first assessed via contrast enhanced CT scan of neck, thorax, abdomen and pelvis at an interim staging after 2 cycles of R-HAD + B / R-HAD. Responding patients (CR or PR) received an additional 2 cycles of therapy. Patients with progressive disease (PD) discontinued study treatment. Before protocol version 3.0 (15.12.2014) became effective in January 2015, treatment was also stopped in case of stable disease (SD) after 2 cycles. From then on, patients with SD after 2 cycles were able to proceed with the treatment at the investigator’s discretion. An end-of-treatment staging via contrast enhanced CT was planned 4–6 weeks after the completion of 4 cycles of therapy. During follow-up, response assessment was planned with contrast enhanced CT scans every three months for two years and every 6 months thereafter for a total of 36 months.

The complete trial protocol is available as supplemental information.

### Statistical methods

TTF was statistically monitored with planned interim analyses for the log-rank statistic using truncated sequential probability ratio test [17]. The study was designed to have 95% power to detect a hazard ratio (HR) of 0.55 for TTF in the R-HAD + B group compared to the R-HAD group (estimated 1/2 years TTF of 50%/30%) with a two-sided significance level of 5%, in which case a median number of 78 events was required, corresponding to randomization of approximately 175 patients during 3.5 years. The maximum number of events was limited to 160 by truncation, which yielded a maximal sample size of approximately 275 patients and a maximal recruiting time of approximately 5.5 years.

As no decision boundary was reached by the end of the trial, the primary comparison between two arms by log-rank test was performed as underrunning analysis of the sequential test, where the adjusted maximum-likelihood estimate for the HR and p-value were calculated correcting for the performed interim analyses. A post hoc power calculation was performed with a significance level of 5% using Schoenfeld method.

Time-to-event outcomes were described with Kaplan-Meier estimates and compared between two treatment arms by log-rank tests without corrections for sequential design. The median follow-up time for TTF was calculated using reverse Kaplan-Meier method. HRs with 95% confidence intervals (CI) were obtained from Cox proportional hazard models, without and with adjustments for MIPI risk score at trial inclusion. Subgroup analyses for the primary outcome were stratified by age, sex, MIPI risk groups, Ki-67 ( ≥ 30% vs. <30%), cytology (pleomorphic/blastoid vs. other), previous lines of therapy, previous high-dose cytarabine, previous ASCT, and progression of disease within 2 years from initial therapy (POD24). Response rates were compared by two-sided Fisher’s exact test. Cumulative incidence of next lymphoma treatment was calculated using cumulative incidence function [18] and compared by Gray’s test, treating death without next lymphoma treatment as a competing event. Maximal grades of Common Terminology Criteria for Adverse Events (CTCAE) Version 4.0 over all cycles of therapy were reported for each category and compared using Fisher’s exact test.

The primary and secondary analyses were performed in a modified intention-to-treat (mITT) population, comprising all randomized patients with confirmed diagnosis of MCL, regardless of the treatment actually received or further protocol violations. Additionally, per-protocol (PP) analyses were performed for the primary outcome, where the mITT patients who received the assigned treatment by randomization and did not stop the treatment prematurely were included and unplanned lymphoma treatment before treatment failure was censored. For safety analysis, patients were evaluated as-treated in the group of treatment started if they received at least one cycle of therapy.

The sample size estimation and the underrunning analysis of the primary outcome were conducted with PEST software version 3. All other statistical analyses were performed using R software version 4.0.4.

## Results

**Fig. 1:**
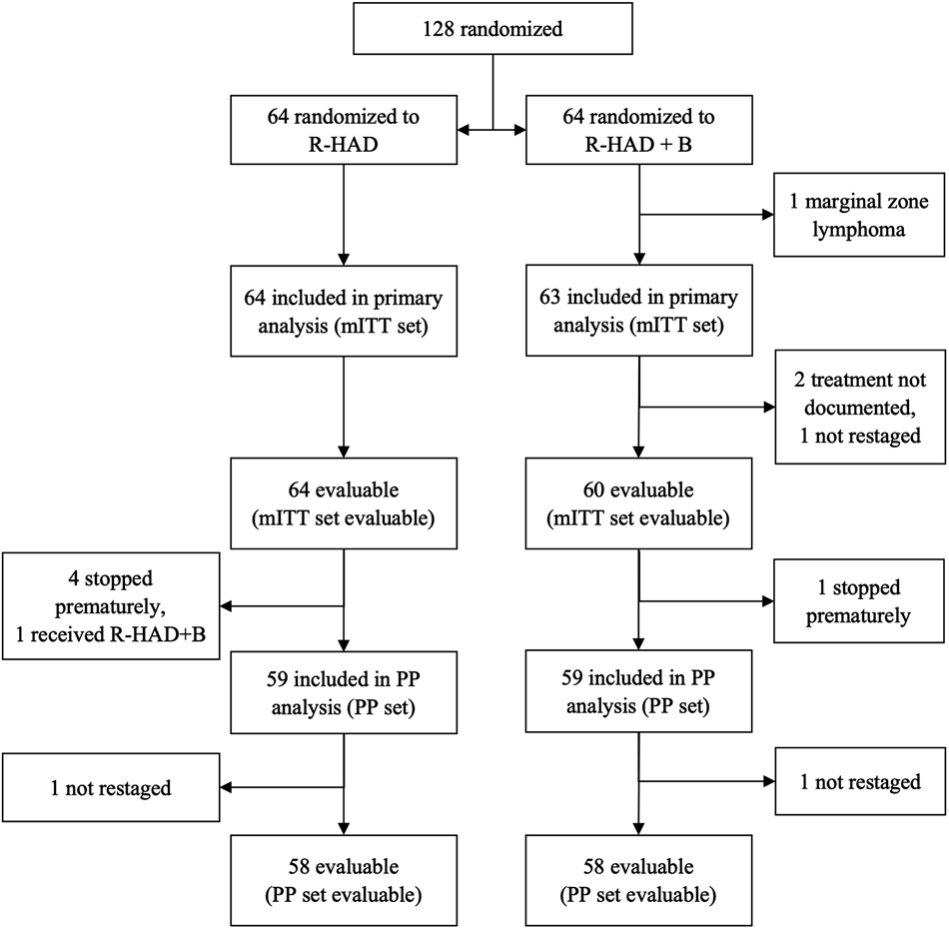
CONSORT flow diagram for the 128 randomized patients. mITT modified intention to treat cohort. PP per protocol cohort.

From May 2012 to December 2016, a total of 128 patients were randomized to either R-HAD + B (*n* = 64) or R-HAD (*n* = 64). Randomization was stopped prematurely due to low recruitment. One patient in the R-HAD + B group was diagnosed with marginal zone lymphoma and excluded from the primary analysis (Fig. 1). The median age of the mITT patients was 70 years (range 41–85). Baseline characteristics of the two groups were fairly balanced (Table 1).

**Table 1:** Baseline characteristics of mITT patients.

Variable	Value	R-HAD (*N* = 64)		R-HAD + B (*N* = 63)	
Study group	LYSA	26	41%	24	38%
	GLSG	38	59%	39	62%
Age (years)	Median, Min-Max	71	41–85	69	41–85
Age	>65	48	75%	41	65%
Sex	Male	51	80%	45	71%
Stage	I	4	6%	5	8%
	II	6	9%	8	13%
	III	5	8%	9	14%
	IV	49	77%	41	65%
ECOG	0	29	45%	24	38%
	1	29	45%	37	59%
	2	6	9%	2	3%
B-symptoms	Present	14	22%	18	29%
LDH ratio to ULN	Median, Min-Max	0.95	0.51–5.79	0.92	0.57–6.74
Hb (g/L)	Median, Min-Max	1.20	0.64–1.66	1.29	0.88–1.56
Leukocytes (10^9^/L)	Median, Min-Max	7.20	0.05–240.60	6.96	1.82–374.76
Thrombocytes (10^9^/L)	Median, Min-Max	150	20–437	161	24–555
Neutr. Granulocytes (10^9^/L)	Median, Min-Max	3.79	0–55.44	3.84	0.38–44.88
Lymphocytes (10^9^/L)	Median, Min-Max	1.94	0.01–233.38	1.41	0.15–363.52
Bone marrow involvement	Present	39	61%	40	63%
Gastrointestinal involvement	Present	9	14%	9	14%
Number of other extranodal involvement	1	6	46% (*n* = 13)	12	86% (*n* = 14)
	2	4	31% (*n* = 13)	2	14% (*n* = 14)
	3	3	23% (*n* = 13)	0	0% (*n* = 14)
Number of Extranodal involvement	Median, Min-Max	1	0–5	1	0–4
MIPI score	Median, Min-Max	6.15	4.96–8.23	6.05	4.92–7.86
MIPI	Low	11	17%	16	25%
	Intermediate	22	34%	24	38%
	High	31	48%	23	37%
Ki-67	High (>=30%)	14	50% (*n* = 28)	16	50% (*n* = 32)
Cytology	Blastoid	6	23% (*n* = 26)	6	19% (*n* = 31)
Previous lines of treatment	1	40	62%	50	79%
	2	17	27%	9	14%
	3	7	11%	4	6%
Previous high-dose cytarabine	Yes	22	34%	24	38%
Previous ASCT	Yes	24	38%	26	41%
Previous remission	Yes	61	95%	61	97%
Primary salvage treatment	Yes	1	2%	2	3%
Time from first diagnosis (years)	Median, Min-Max	3.70	0.09–14.2	3.89	0–11.11
Time from last relapse/progression (days)	Median, Min-Max	27	4–1504	31	2–1136

Baseline characteristics of mITT patients randomized to R-HAD or R-HAD + B.

*mITT* modified intention to treat population, *ECOG* Eastern cooperative oncology group performance status, *LDH* lactate dehydrogenase, *ULN* upper limit of norm, *Hb* Hemoglobin, *Neutr.* neutrophile, *MIPI* Mantle cell lymphoma international prognostic index, *ASCT* autologous stem cell transplant.

### Response rates

At the end of induction, 38 (63%) patients from the R-HAD + B group and 29 (45%) patients from the R-HAD group achieved an overall response (ORR; *p* = 0.049, Table 2). 3 patients from the R-HAD + B group were not staged after induction. More patients achieved a CR/CRu in the R-HAD + B group than in the R-HAD group (42% vs. 19%, p = 0.0062). 22% (*n* = 14) and 30% (*n* = 19) of patients treated with R-HAD + B and R-HAD achieved only a SD after two cycles, of whom 11 and 6 patients stopped treatment. All patients with PD after two cycles (R-HAD + B: *n* = 4, R-HAD: *n* = 13) stopped the study treatment.

**Table 2:** Response rates.

	R-HAD (*N* = 64)		R-HAD + B (*N* = 60)*		*P* value
Complete remission (CR)	8	12%	17	28%	0.043
Complete remission (CR, CRu)	12	19%	25	42%	0.0062
Overall response (CR, CRu, PR)	29	45%	38	63%	0.049

Response rates at end of induction for patients treated with R-HAD and R-HAD + B* 3 patients from R-HAD + B group were excluded from the analysis because of missing staging results during induction.

*CR* complete remission, *CRu* complete remission unconfirmed, *PR* partial remission.

Taken together, 68% vs. 55% of all patients received a full 4 cycles of immunochemotherapy in the R-HAD + B vs. R-HAD groups, respectively (Supplementary Table S1).

### Time-to-event outcomes

By the data cut-off, the median follow-up for mITT patients was 41 months. The median TTF in the R-HAD + B group (12.0 months) was longer than in the R-HAD group (2.6 months, Fig. S1). The underrunning analysis corrected for interim analyses revealed a *p* = 0.045 and a hazard ratio for R-HAD + B vs. R-HAD of 0.68. A post hoc power of only 51.3% was estimated to detect such a hazard ratio with 107 available events of treatment failure (51 in R-HAD + B group, 56 in R-HAD group) and 5% significance level.

**Fig. 2:**
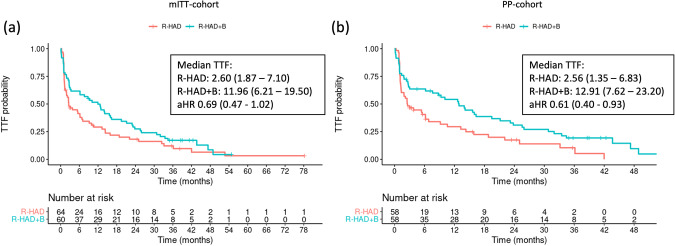
Time to treatment failure for R-HAD + B vs. R-HAD. Kaplan-Meier plots for time to treatment failure in the (**a**) modified intention to treat (mITT) and the (**b**) per protocol (PP) population. aHR Adjusted hazard ratio.

After the cut-off date, an unplanned data update was performed and one additional event of treatment failure in the R-HAD + B group was documented. All following analyses were performed with this final dataset. This event resulted in a MIPI-adjusted HR (aHR) of 0.69 (95%CI 0.47–1.02; median TTF 12 vs. 2.6 months, underrunning log-rank p = 0.089). A slightly larger treatment effect of R-HAD + B was found in the per-protocol analysis, where the median TTF in the R-HAD + B group was 13.0 months compared to 2.6 months in the R-HAD group (aHR 0.61; 0.40–0.93; Fig. 2a, b).

**Fig. 3:**
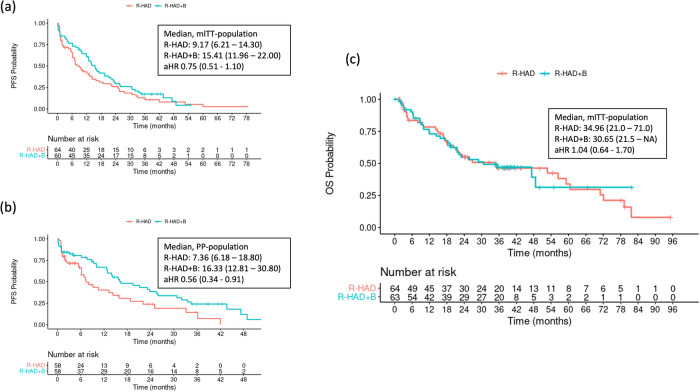
Progression free and overall survival for R-HAD + B vs. R-HAD. Kaplan-Meier plots for (**a**) progression free survival (PFS) in the modified intention to treat (mITT) population; (**b**) PFS in the per protocol population (PP) and (**c**) overall survival (OS) in the mITT population. aHR Adjusted hazard ratio.

The PFS was numerically longer in patients in the R-HAD + B group than in the R-HAD group (median 15.4 vs. 9.2 months, aHR 0.75 (0.51–1.10), Fig. 3a). A statistically significant treatment effect of R-HAD + B was observed in the per-protocol analysis (median PFS 16.3 vs. 7.4 months, aHR 0.56, 95%CI 0.34–0.91, Fig. 3a, b).

Among responding patients, the median DOR was 20.7 and 13.5 months in the R-HAD + B and R-HAD groups, respectively (aHR 0.74, 0·41–1.33). Patients treated in their first relapse had a longer DOR (median: 23.2 and 19.3 months; *p* = 0.57) than in later relapses (median: 9.2 and 4.7 months; *p* = 0.79), without significant differences between treatment groups.

12 patients from the R-HAD + B group and 19 patients from the R-HAD group received next line treatment (12-month probability: 18% vs. 28%; 24-month probability: 20% vs. 32%; *p* = 0.17, Fig. S2). The probability of death without a next lymphoma treatment was similar between two groups.

No difference in OS was observed for R-HAD + B vs. R-HAD (median: 30.7 vs. 35.0 months, aHR 1.04, 0.64–1.70, Fig. 3c).

### Subgroup analyses

**Fig. 4:**
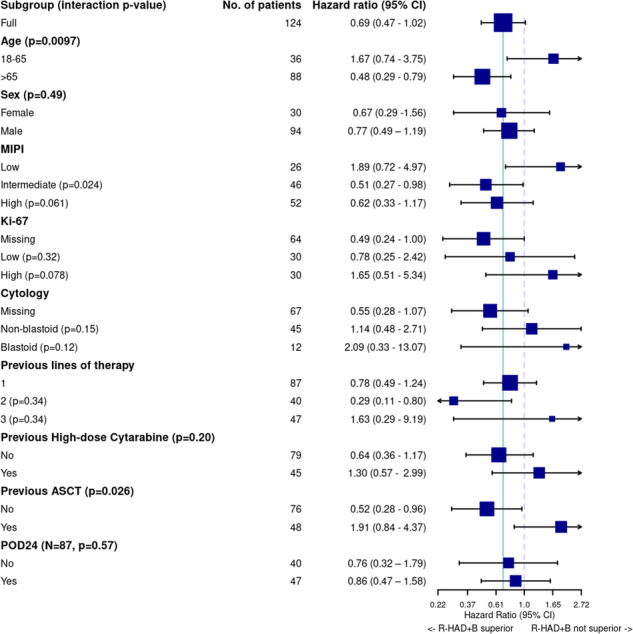
Forest plot of time to treatment failure (TTF) in subgroups of modified intention to treat (mITT) patients, adjusted for MIPI (except MIPI subgroups). *P* values are interaction *p* values, indicating possible effect differences across subgroups. The solid blue vertical line marks the treatment effect in the whole cohort. The dashed vertical line marks HR = 1. CI confidence interval, MIPI Mantle cell lymphoma international prognostic index, ASCT autologous stem cell transplant, POD24 progression of disease within 24 months of initial treatment.

Prolonged TTF was observed in the R-HAD + B group compared to the R-HAD group in the subgroups of elderly patients >65 years (median 12.8 vs. 2.5 months, aHR 0.48, 95%CI 0.29–0.79; interaction p-value for age >65 / 18–65 years: 0.0097), patients without previous high-dose cytarabine treatment (median 16.0 vs. 2.2 months, aHR 0.64 (0.36–1.17), interaction *p* value HD-cytarabine yes/no: 0.20) and patients without previous ASCT (median 12.7 vs. 2.2 months, aHR 0.52 (0.28–0.96), interaction p-value ASCT yes/no: 0.026, Fig. 4).

### Toxicity

Most patients (98% in R-HAD, 100% in R-HAD + B) experienced at least one adverse event (grade 1 to 4) during the treatment. Hematological toxicities remained the most frequent adverse events in both groups, whereas slightly higher rates of grade 3 or 4 thrombocytopenia (76% vs. 62%), neutropenia (65% vs. 49%), leukocytopenia (57% vs. 38%), and lymphocytopenia (56% vs. 37%) occurred in the R-HAD + B group than in the R-HAD group (Table 3). Dose reductions were necessary in 17 and 6 occasions in the R-HAD + B and R-HAD group, mostly related to cytarabine. In the R-HAD + B group, toxicity-related dose reductions were associated with cytarabine in 12 and bortezomib in 7 cases (Supplementary Table S2). No treatment related causes of death were reported. Five patients in the R-HAD + B and four patients in the R-HAD group died without receiving further treatment lines after study therapy. In R-HAD + B, two patients died due to progressive disease, two due to secondary malignancies (one glioma and one carcinoma) and one was lost to follow-up with the cause of death unknown. In R-HAD, three patients died due to progressive disease and one patient died because of a preexisting small cell pulmonary carcinoma. Of the 61 patients that died after receiving subsequent lines of lymphoma therapy, the majority (*n* = 37) died due to lymphoma progression (Supplementary Table S3).

**Table 3:** Observed grade ≥3 toxicity over all cycles (in >10% of patients of all grades in any study arm).

	R-HAD (*N* = 63)		R-HAD + B (*N* = 63)		*P* value
Any Adverse Events	62	98%	63	100%	>0.99
Grade 3 or 4 Events					
Thrombocytopenia	39	62%	48	76%	0.12
Neutropenia	31	49%	41	65%	0.10
Leucocytopenia	24	38%	36	57%	0.049
Lymphocytopenia	23	37%	35	56%	0.049
Anemia	17	27%	13	21%	0.53
Infection	14	22%	10	16%	0.50
Fever	2	3%	3	5%	>0.99
Diarrhea	1	2%	2	3%	>0.99
Constipation	0	0%	1	2%	>0.99
Peripheral_neuropathy	0	0%	1	2%	>0.99
Mood alteration/ depre.	1	2%	0	0%	>0.99
Myalgia / Arthralgia	1	2%	0	0%	>0.99
Fatigue	1	2%	1	2%	>0.99
Hemorrhage	3	5%	0	0%	0.24
Pulmonary function	4	6%	2	3%	0.68
Arrhythmia	0	0%	2	3%	0.50
Creatinine increased	2	3%	0	0%	0.50
GOT/GPT increased	1	2%	2	3%	>0.99
Bilirubin increased	0	0%	1	2%	>0.99

Observed Grade ≥3 toxicity. Only toxicity that occurred in >10% of patients (grade 1–5) in any study arm is reported here.

## Discussion

Bortezomib has previously shown efficacy in r/r MCL e.g. in combination with bendamustine-rituximab (2-year PFS 47%) [19] or in combination with CHOP chemotherapy (median PFS 16.5 months) [20]. In this trial we add further evidence that bortezomib in combination with R-HAD immunochemotherapy can be effective in r/r MCL patients, at least in specific subsets. Several observations of our analysis support this hypothesis: First, even though the difference in TTF between R-HAD + B and R-HAD lacks statistical significance in the final analysis in the mITT cohort due to limited power because of the early termination of this trial, the numerical difference is notable (12 vs. 2.6 months). Additionally, the last pre-planned interim analysis before the early closure of this trial showed a statistically significant treatment effect of bortezomib for the primary endpoint TTF (Fig. S1). TTF and PFS were also significantly increased for R-HAD + B over R-HAD in the per protocol set (Fig. 2). Second, R-HAD + B was superior to R-HAD regarding response rates, with OR and CR/CRu rates of 63% vs. 45% (*p* = 0.049) and 42% vs. 19% (*p* = 0.0062), respectively.

Interestingly, in the subgroup analyses, bortezomib seemed to improve TTF especially in the more vulnerable patient populations: Patients >65 years as well as patients without previous ASCT or HD-cytarabine treatment, whereas patients ≤65 years or after intensive treatment regimens seemed to benefit less (Fig. 4).

These observations fall in line with previously published data in untreated MCL patients: Bortezomib has been shown to significantly improve survival of elderly MCL patients in a pivotal phase 3 trial of R-CHOP vs. VR-CAP (bortezomib, rituximab, cyclophosphamide, doxorubicin, prednisolone) [11], whereas studies in younger patients, combining bortezomib with various intensive treatment regimens yielded more conflicting results [21].

The median DOR for R-HAD + B of 23.2 months in first relapse seems favorable in context to what has been reported in other r/rMCL trials except BTK inhibitors [22–24]. Ibrutinib, which, with a median DOR of 35.6 months in first relapse, was widely considered standard of care in r/rMCL until recently [25], is currently moving into first-line therapy regimens for younger and elderly patients [9, 10]. Taken together, our data implies a possible role of bortezomib-based combination strategies in first relapse, if ibrutinib is not an option.

Our study is hampered by a few limitations: When the trial was planned, the MIPI was not yet established and stratification was done according to IPI, even though it does not adequately predict outcome in MCL patients [4]. To account for that, analyses were also performed adjusted for MIPI score.

Second, due to slow enrollment, the trial was terminated prematurely with only 128 randomized patients, falling short of the planned 175 participants (maximum 275 participants). Consequently, the trial lacked adequate patient numbers and events rendering it insufficiently powered to reliably detect differences between both treatment arms. Furthermore, compared to other prospective trials of rituximab-chemotherapy combinations in r/r MCL, the primary endpoint, median TTF, in the R-HAD arm was short: historic prospective data showed a median PFS of 8 months for rituximab + fludarabine, cyclophosphamide and mitoxantrone (R-FCM) [13] and a median PFS of around 17 months for bendamustine-rituximab (BR) [26], compared to a median TTF of only 2.60 months in our trial. Of note, median PFS times for R-HAD and R-HAD + B (9.2 and 15.4 months) were more comparable and the very short TTF might be partly owed to an overambitious trial design: Treatment failure was defined as SD or PD with patients having to discontinue the treatment if SD was reported at the interim staging after only two cycles of therapy. This was changed in protocol version 3.0 (15.12.2014), from then on allowing patients with SD after cycle 2 to continue the study treatment at the investigator’s discretion. However, only few patients were included after protocol version 3.0 came into effect before the early closure of the trial. Therefore, in addition to TTF, PFS and DOR should be carefully appreciated when evaluating efficacy. Lastly, maintenance therapy was not applied systematically since the trial was planned before rituximab-maintenance was shown to improve PFS and OS in MCL [5].

Despite those limitations, certain key insights can be discerned: Our data adds further evidence, suggesting that HD-cytarabine alone is insufficient to induce meaningful responses in MCL. Similarly, another trial of the Nordic Lymphoma Group in younger, high risk, first line MCL patients testing a high dose cytarabine + ASCT containing regime was prematurely stopped during the safety and efficacy run-in phase because it failed in 4 out of 5 patients (3 were non-responders and one progressed after an initial response) [27]. Furthermore, most first-line trials showing favorable results for the addition of HD-cytarabine also included alkylating agents, anthracyclines or vinca alcaloids [5–7, 28, 29].

### Safety / toxicity

Apart from peripheral sensory neuropathy, bortezomib is known to increase hematological toxicity. Robak and colleagues reported grade ≥3 thrombocytopenia for VR-CAP vs. R-CHOP in 57% vs. 6% of patients, respectively [30]. In our protocol, we scheduled the addition of bortezomib only on day 1 and 4 to reduce hematological and neurological toxicity. With this regimen, grade 3–4 hematological toxicity, albeit high, was similar between both treatment arms and largely attributable to the chemotherapeutic backbone. Only leukocytopenia and lymphocytopenia was significantly more frequent in R-HAD + B than in R-HAD. Thrombocytopenia grade ≥3 occurred in 76% and 62% of patients, respectively, and this difference was not statistically significant. Further, this increase in toxicity might be partly explained by a higher exposure to immunochemotherapy in the R-HAD + B group: More patients in the R-HAD + B group received a full 4 cycles of immunochemotherapy compared to the R-HAD group.

The rate of thrombocytopenia in our trial is comparable to what was observed after the addition of HD-cytarabine to first line therapy in the MCL younger trial [7] and is distinctly higher than what was reported in other trials involving bortezomib monotherapy in r/r MCL. Grade ≥3 thrombocytopenia was shown in 11% of r/r MCL [14] after bortezomib monotherapy and in 21.7% after bortezomib-CHOP [20], suggesting that the thrombocytopenia might be largely owed to HD-cytarabine in our trial.

Peripheral neuropathy (PNP) was mostly grade 1–2 and not significantly different between both treatment arms. Taken together, bortezomib given on day 1 and 4 in combination with R-HAD was well tolerated and without major or unexpected additional toxicities.

In conclusion, bortezomib in combination with HD-cytarabine is well tolerated and our trial provides further hypothesis-generating data, suggesting efficacy in relapsed and refractory MCL patients. HD-cytarabine alone might be insufficient as a chemotherapeutic backbone and a combination with other chemotherapeutic agents like alkylators or anthracyclines might be warranted. Further investigations are needed to determine the efficacy of bortezomib as salvage therapy, especially now, that BTK-inhibitors are advancing into first-line MCL therapy.

### Supplementary information

Supplemental Information

Supplemental Material

## Data Availability

Anonymized clinical data and the statistical plan underlying the analysis might be shared upon request to the corresponding author on the basis of scientific collaboration. The study protocol is available as supplemental material.
