# Supplementary material for: The addition of bortezomib to rituximab, high-dose cytarabine and dexamethasone in relapsed or refractory mantle cell lymphoma—a randomized, open-label phase III trial of the European mantle cell lymphoma network
Source: Leukemia. 2024 Apr 27;38(6):1307–14. doi: 10.1038/s41375-024-02254-2 (PMC11147755; doi:10.1038/s41375-024-02254-2)
Supplement: Supplementary file 1 — Supplemental Information [file 41375_2024_2254_MOESM1_ESM.docx]

**Supplemental Information**

**Supplemental Methods:**

**Inclusion criteria:**

- Confirmed pathological diagnosis of MCL according to WHO classification.
- Relapse or progression following 1 to 3 prior lines of anti-neoplastic standard therapy. Therapy in remission after initial induction like intensified chemotherapy for stem cell separation followed by myeloablative therapy or any kind of maintenance therapy is classified as one line of therapy with the induction therapy.
- If Rituximab was part of prior induction treatment, documented time to progression must be at least 12 weeks after this particular regimen.
- If high-dose Ara-C was part of prior treatment, documented time to progression must be at least 6 months after this particular regimen.
- Patients relapsed after autologous stem cell transplantation or not appropriate for myeloablative treatment.
- At least 1 measurable or assessable site of disease; in case of bone marrow infiltration only, bone marrow aspiration/ biopsy is mandatory for all staging evaluations.
- age ≥ 18 years
- ECOG/WHO Performance Score 0-2 unless lymphoma related.
- The following laboratory values at screening, unless lymphoma related:
- Absolute neutrophil count (ANC) ≥1500 cells/µL
- Platelets ≥100,000 cells/µL
- Transaminases (AST and ALT) ≤3 x upper limit of normal (ULN)
- Total bilirubin ≤2 x ULN
- Creatinine ≤2 mg/dL or calculated creatinine clearance ≥50 mL/min
- Toxic effects of previous therapy or surgery resolved to NCI CTC grade 2 or better.
- Premenopausal fertile females must agree to use a highly effective method of birth control for the duration of the therapy. A highly effective method of birth control is defined as those which result in a low failure rate (i.e. less than 1% per year) when used consistently and correctly such as implants, injectables, combined oral contraceptives, some IUDs, sexual abstinence or vasectomised partner.
- Men must agree not to father a child for the duration of therapy and must agree to advice a female partner to use a highly effective method of birth control.
- Written informed consent before performance of any study-related procedure.

**Exclusion criteria:**

- Treatment within another clinical trial within 30 days before trial entry or planed during this trial
- Anti-neoplastic (including radiation and antibody treatment) or experimental therapy within 4 weeks before planed Day 1 of Cycle 1 (Nitrosoureas within 6 weeks) or radioimmunoconjugates or toxin immunoconjugates such as Ibritumomab tiuxetan (Zevalin™) or Tositumomab (Bexxar®) within 12 weeks before planed Day 1 of Cycle 1
- Known hypersensitivity to Rituximab, boron or mannitol. - Active malignancy other than MCL within 5 years before Day 1 of Cycle 1, with the exception of complete resection of basal cell carcinoma, squamous cell carcinoma of the skin, or in situ malignancy.
- Active systemic infection requiring treatment.
- HIV, hepatitis B or C
- Patient has > grade 2 peripheral sensory neuropathy or neuropathic pain defined by the NCI Common Terminology Criteria for Adverse Events (CTCAE).
- Symptomatic degenerative or toxic encephalopathy
- Serious medical condition (such as severe hepatic impairment, pericardial disease, acute diffuse infiltrative pulmonary disease, systemic infections etc.) or psychiatric illness likely to interfere with participation in this clinical study
- Female subject is pregnant or breast-feeding (pregnancy testing is mandatory for premenopausal women).

**Response Evaluationa according to the International Workshop to Standardize Response Criteria for Non-Hodgkin’s Lymphoma:**

Complete response (CR): Complete disappearance of all detectable clinical and radiographic evidence of disease and disappearance of all disease-related symptoms if present before therapy, and normalisation of those biochemical abnormalities (e.g. lactate dehydrogenase (LDH) definitely assignable to NHL.
All lymph nodes and nodal masses must have regressed to normal size (<1.5 cm in their greatest transverse diameter for nodes > 1.5 cm before therapy). Previously involved nodes that were 1.1 to 1.5 cm in their greatest transverse diameter before treatment must have decreased to 1 cm in their greatest transverse diameter after treatment, or by more than 75% in the sum of the products of the greatest diameters (SPD).

The spleen, if considered to be enlarged before therapy on the basis of a CT scan, must have regressed in size and must not be palpable on physical examination. However, no normal size can be specified because of the difficulties in accurately evaluating splenic and hepatic size. For instance, spleens thought to be of normal size may contain lymphoma, whereas an enlarged spleen may not necessarily reflect the presence of lymphoma but variations in anatomy, blood volume, the use of hematopoietic growth factors, or other causes. Any macroscopic nodules in any organs detectable on imaging techniques should no longer be present. Similarly, other organs considered to be enlarged before therapy due to involvement by lymphoma, such as liver and kidneys, must have decreased in size.

If the bone marrow was involved by lymphoma before treatment, the infiltrate must be cleared on repeat bone marrow aspirate and biopsy of the same site. The sample on which this determination is made must be adequate (>20 mm biopsy core). Flow cytometric, molecular, or cytogenetic studies arenot considered part of routine assessment to document persistent disease at the present time.

**CR/unconfirmed (CRu**) includes those patients who achieve a complete disappearance of all clinical symptoms or organ involvement, but with one or more of the following features:
A residual lymph node mass greater than 1.5 cm greatest transverse diameter that has regressed by more than 75% in the SPD. Individual nodes that were previously confluent must have regressed by more than 75% in their SPD compared with the size of the original mass.

Indeterminate bone marrow (increased number or size of aggregates without cytological or architectural atypia)

Partial remission (PR): 50% decrease in SPD of the six largest dominant nodes or nodal masses. These nodes or masses should be selected according to the following features:

- they should be clearly measurable in at least two perpendicular dimensions,
- they should be from as completed disparate regions of the body as possible, and
- they should include mediastinal and retroperitoneal areas of disease whenever these sites are involved.
- No increase in the size of the other nodes, liver, or spleen.
- Splenic and hepatic nodules must regress by at least 50% in the SPD.
- With the exception of splenic and hepatic nodules, involvement of other organs is considered assessable and not measurable disease.
- Bone marrow assessment is irrelevant for determination of a PR because it is assessable and not measurable disease; however, if positive, the cell type should be specified in the report and preferably confirmed by immunohistochemistry.
- No new sites of disease.

Stable disease/No change (SD): less than a PR (see above) but is not progressive disease (see below).

Relapsed disease (after CR, CRu):

- Appearance of any new lesion or increase by > 50% in the size of previously involved sites.
- >50% increase in greatest diameter of any previously identified node greater than 1 cm in its short axis or in the SPD of more than one node.

Progressive disease (after PR, non-responders):

- >50% increase from nadir in the SPD of any previously identified abnormal node for PRs or non-responders.
- Appearance of any new lesion during or at the end of therapy

**Supplemental Tables**

Table S1: Number of cycles

| **No. of cycles** | **R-HAD (N=64)** | | **R-HAD+B (N=63)** | |
| --- | --- | --- | --- | --- |
| **0** | 0 | 0% | 2 | 3% |
| **1** | 3 | 5% | 7 | 11% |
| **2** | 24 | 38% | 10 | 16% |
| **3** | 2 | 3% | 1 | 2% |
| **4** | 35 | 55% | 43 | 68% |

Table S1: Number of immuno-chemotherapy cycles by study arm

Table S2: Toxicity-related dose reductions

| **Cycle** | **Toxicity-related dose reduction** | **R-HAD** | **R-HAD+B** |
| --- | --- | --- | --- |
| **2** | **Yes** | 3 (n=59) | 4 (n=54) |
|  | Ara-C | 3 | 4 |
|  | Bortezomib | NA | 1 |
|  | Other | 0 | 0 |
| **3** | **Yes** | 2 (n=36) | 7 (n=44) |
|  | Ara-C | 2 | 4 |
|  | Bortezomib | NA | 3 |
|  | Other | 0 | 2 |
| **4** | **Yes** | 1 (n=34) | 6 (n=43) |
|  | Ara-C | 1 | 4 |
|  | Bortezomib | NA | 3 |
|  | Others | 0 | 0 |

Table S2: Toxicity-related dose reductions for the R-HAD and R-HAD+B groups in each cycle of immunochemotherapy.

Table S3: Causes of death

|  | **Number** | **Comment** |
| --- | --- | --- |
| **Death due to lymphoma after induction (R-HAD/R-HAD+B)** | 5 (3 / 2) |  |
| **Death in remission (R-HAD+B / R-HAD)** | 4 (3 / 1) | R-HAD+B: 1x lost to follow-up after 1 cycle of R-HAD+B; 1x glioma; 1x carcinoma  R-HAD: SCLC (preexisting) |
| **Death after subsequent line of therapy** | 61 | Lymphoma: 37  Infection: 8  TRM after AlloSCT: 8  Other: 4  Unknown: 4 |

Table S3: Causes of death after therapy with R-HAD or R-HAD+B or after subsequent line(s) of lymphoma therapy. SCLC small cell lung cancer. TRM treatment related mortality. AlloSCT allogenic stem cell transplantation.

**Supplemental Figures:**

Figure S1:


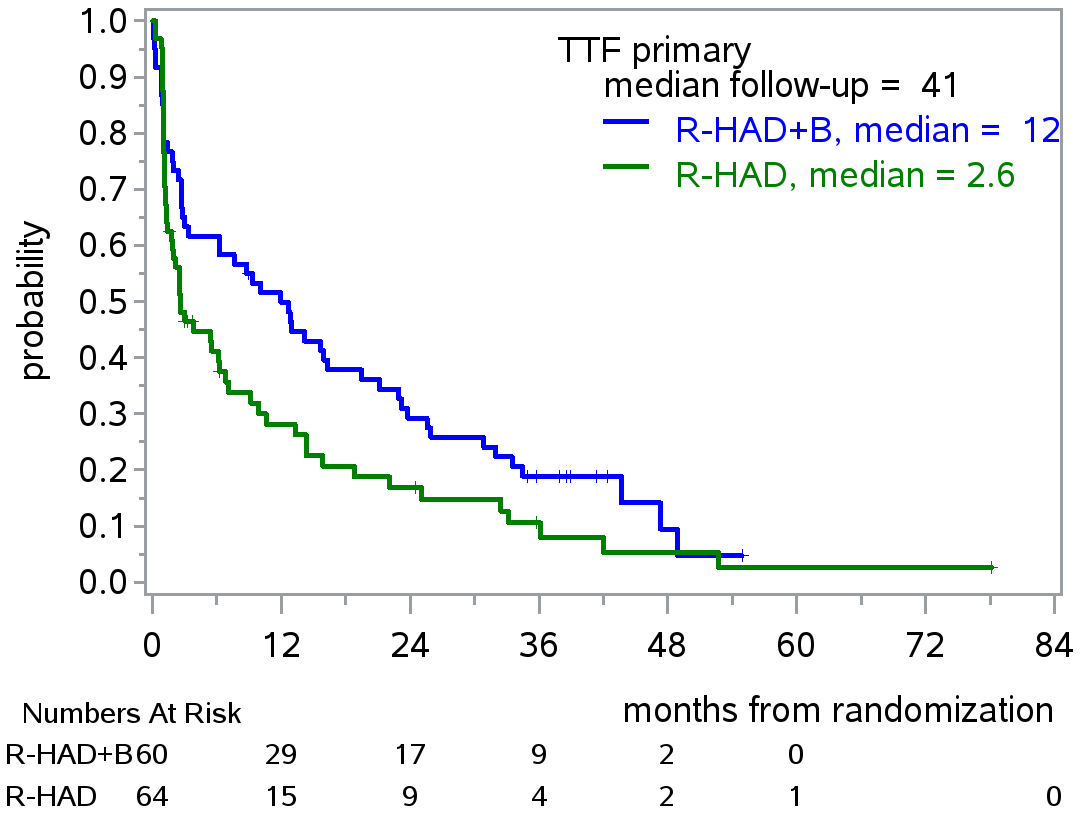


Figure S1: Time to treatment failure (TTF) in the mITT of the pre-planned primary underrunning analysis

Figure S2:


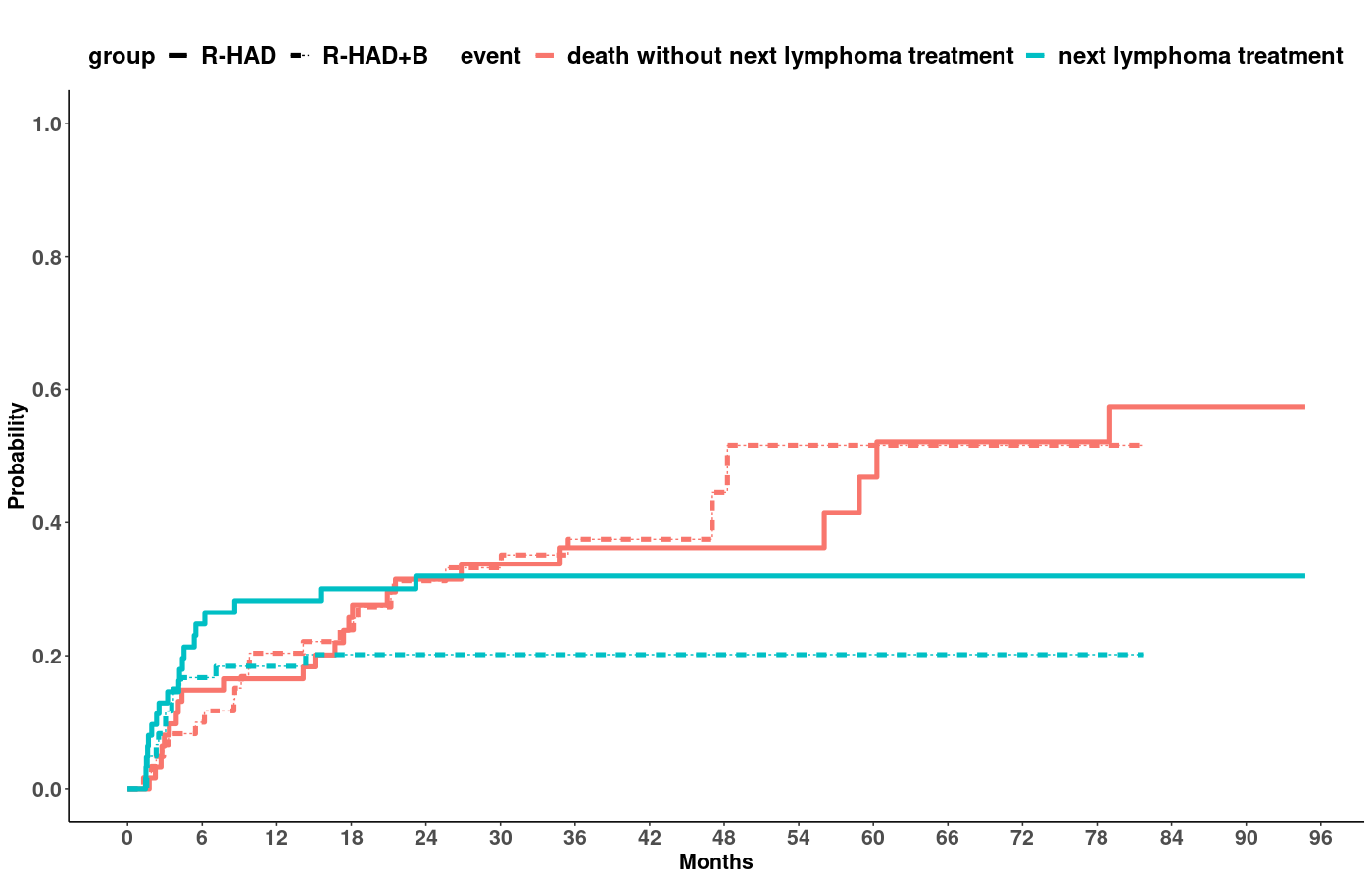


Figure S2: Time to next lymphoma treatment in the mITT population. The continuous lines represent patients randomized to R-HAD, the dotted lines represent patients randomized to R-HAD+B.
