# Supplementary material for: The addition of bortezomib to rituximab, high-dose cytarabine and dexamethasone in relapsed or refractory mantle cell lymphoma—a randomized, open-label phase III trial of the European mantle cell lymphoma network
Source: Leukemia. 2024 Apr 27;38(6):1307–14. doi: 10.1038/s41375-024-02254-2 (PMC11147755; doi:10.1038/s41375-024-02254-2)
Supplement: Supplementary file 2 — Supplemental Material [file 41375_2024_2254_MOESM2_ESM.pdf]

EFFICACY AND SAFETY OF RITUXIMAB, HIGH-DOSE ARA-C AND DEXAMETHASONE (R-HAD) ALONE OR IN COMBINATION WITH BORTEZOMIB IN PATIENTS WITH RELAPSED OR REFRACTORY MANTLE CELL LYMPHOMA

A RANDOMIZED PHASE III TRIAL OF THE *EUROPEAN MCL NETWORK*

Writing committee: Dreyling M, Ribrag V, Weigert O, Hoster E, Gressin R, Montserrat E, Walewski J, Geisler C, Shpilberg O, Hiddemann W

Sponsor:

Klinikum der Ludwig-Maximilians-Universität München
Klinikum der Universität München
Marchioninistraße 15
81377 München
Kaufmännischer Direktor
Dipl. – Kfm. Gerd Koslowski
Tel.: +49-89-4400-72001,
Fax: +49-89-4400-72002

Person authorised by the sponsor:
Prof. Dr. Martin Dreyling, Tel.: +49-89-4400-72202, Fax: +49-89-4400-72201

Data Center:
European MCL Network
Dr. M. Unterhalt
Klinikum der Universität München
Dept. of Medicine III
- Study Center -
Marchioninistraße 15
D-81377 Munich / GERMANY
Phone: +49-89-4400-74900 / -74901
Fax: +49-89-4400-77900 / -77901

e-mail: studyce@med.uni-muenchen

EudraCT-Nr.: 2005-005144-62
Prüfplancode Sponsor: MCL2005-01

Protocol Version 3.0 FINAL
Date: 15.12.2014

Coordinating investigator, Germany:
Prof. Dr. M. Dreyling
Klinikum der Universität München
Dept. of Medicine III
Marchioninistraße 15
D-81377 Munich / GERMANY
Phone: +49-89-4400-72202
Fax: +49-89-4400-72201
e-mail: martin.dreyling@med.uni-muenchen.de

Coordinating investigator, France:
Dr. V. Ribrag
Institut Gustave Roussy
Dept. of Medicine
39 rue C Desmoulins
94805 Villejuif / FRANCE
Phone: +33-1-42-11-43-21
Fax: +33-1-42-11-43-47
e-mail: ribrag@igr.fr

Since this trial is done in an international cooperation, for each national site a coordinating investigator has to be listed. The list of coordinating investigators has to be expanded when the trial is established in an additional national site. This will be done by an amendment which will be handled as not substantial amendment for the other sites.

The content of this document is strictly confidential and may not be copied or made accessible to third parties without written consent of the Hospital of the University of Munich

SIGNATURE OF SPONSOR, COORDINATING INVESTIGATORS AND RESPONSIBLE STATISTICIAN

____

M. Dreyling
(Persons authorised by the sponsor,
coordinating investigator, Germany)

____

E. Hoster (responsible statistician)

____

V. Ribrag (coordinating investigator, France)

II SYNOPSIS

Title

Efficacy and safety of Rituximab, high-dose Ara-C and dexamethasone (R-HAD) alone or in combination with Bortezomib in patients with relapsed or refractory mantle cell lymphoma

Trial design

Prospective, randomized, multicenter phase III trial

Trial endpoints: - primary

Time to treatment failure

- secondary

Complete and overall response rate, progression-free survival, duration of remission, time to next lymphoma treatment, overall survival, safety and tolerability of Rituximab, high-dose Ara-C and dexamethasone alone or in combination with Bortezomib

Treatment

Treatment course will be repeated in 3-week intervals (day 22 +/- 3 days):

Rituximab, 375 mg/m² IV, d1

Ara-C 2000 mg/m² (patients >65 years or s/p myeloablative treatment: 1000 mg/m²) IV, d 2 and 3

Dexamethasone 40 mg PO d 1- 4

± Bortezomib 1.5 mg/m² SC, d 1 and 4

After 2 treatment cycles a midterm staging will be performed. Responders to induction therapy will receive 2 additional treatment cycles in case of adequate tolerability. In case of stable disease, patients may proceed with the treatment at the investigator's discretion.

Inclusion criteria

- Confirmed pathological diagnosis of MCL according to WHO classification.
- Relapse or progression following 1 to 3 prior lines of anti-neoplastic standard therapy. Therapy in remission after initial induction like intensified chemotherapy for stem cell separation followed by myeloablative therapy or any kind of maintenance therapy is classified as one line of therapy with the induction therapy.
- If Rituximab was part of prior induction treatment, documented time to progression must be at least 12 weeks after this particular regimen.
- If high-dose Ara-C was part of prior treatment, documented time to progression must be at least 6 months after this particular regimen.
- Patients relapsed after autologous stem cell transplantation or not appropriate for myeloablative treatment.
- At least 1 measurable or assessable site of disease; in case of bone marrow infiltration only, bone marrow aspiration/ biopsy is mandatory for all staging evaluations.
- age ≥ 18 years
- ECOG/WHO Performance Score 0-2 unless lymphoma related.
- The following laboratory values at screening, unless lymphoma related:
    - Absolute neutrophil count (ANC) ≥1500 cells/µL
    - Platelets ≥100,000 cells/µL
    - Transaminases (AST and ALT) ≤3 x upper limit of normal (ULN)
    - Total bilirubin ≤2 x ULN
    - Creatinine ≤2 mg/dL or calculated creatinine clearance ≥50 mL/min
- Toxic effects of previous therapy or surgery resolved to NCI CTC grade 2 or better.
- Premenopausal fertile females must agree to use a highly effective method of birth control for the duration of the therapy. A highly effective method of birth control is defined as those which result in a low failure rate (i.e. less than 1% per year) when used consistently and correctly such as implants, injectables, combined oral contraceptives, some IUDs, sexual abstinence or vasectomised partner.

	- Men must agree not to father a child for the duration of therapy and must agree to advice a female partner to use a highly effective method of birth control.<br>- Written informed consent before performance of any study-related procedure.
Exclusion criteria	- Treatment within another clinical trial within 30 days before trial entry or planed during this trial<br>- Anti-neoplastic (including radiation and antibody treatment) or experimental therapy within 4 weeks before planed Day 1 of Cycle 1 (Nitrosoureas within 6 weeks) or radioimmunoconjugates or toxin immunoconjugates such as Ibritumomab tiuxetan (Zevalin™) or Tositumomab (Bexxar®) within 12 weeks before planed Day 1 of Cycle 1<br>- Known hypersensitivity to Rituximab, boron or mannitol.<br>- Active malignancy other than MCL within 5 years before Day 1 of Cycle 1, with the exception of complete resection of basal cell carcinoma, squamous cell carcinoma of the skin, or in situ malignancy.<br>- Active systemic infection requiring treatment.<br>- HIV, hepatitis B or C<br>- Patient has $\geq$ grade 2 peripheral sensory neuropathy or neuropathic pain defined by the NCI Common Terminology Criteria for Adverse Events (CTCAE).<br>- Symptomatic degenerative or toxic encephalopathy<br>- Serious medical condition (such as severe hepatic impairment, pericardial disease, acute diffuse infiltrative pulmonary disease, systemic infections etc) or psychiatric illness likely to interfere with participation in this clinical study<br>- Female subject is pregnant or breast-feeding (pregnancy testing is mandatory for premenopausal women).
Number of study centers	all centers of the *European MCL Network* may apply<br>expected number of participating sites: 100-120
Number of patients	approximately 175 patients, maximum of 275 patients
Duration of recruitment	approximately 3.5 years, maximum of 5.5 years<br>first patient in: 2nd quarter 2012

III TRIAL DESIGN

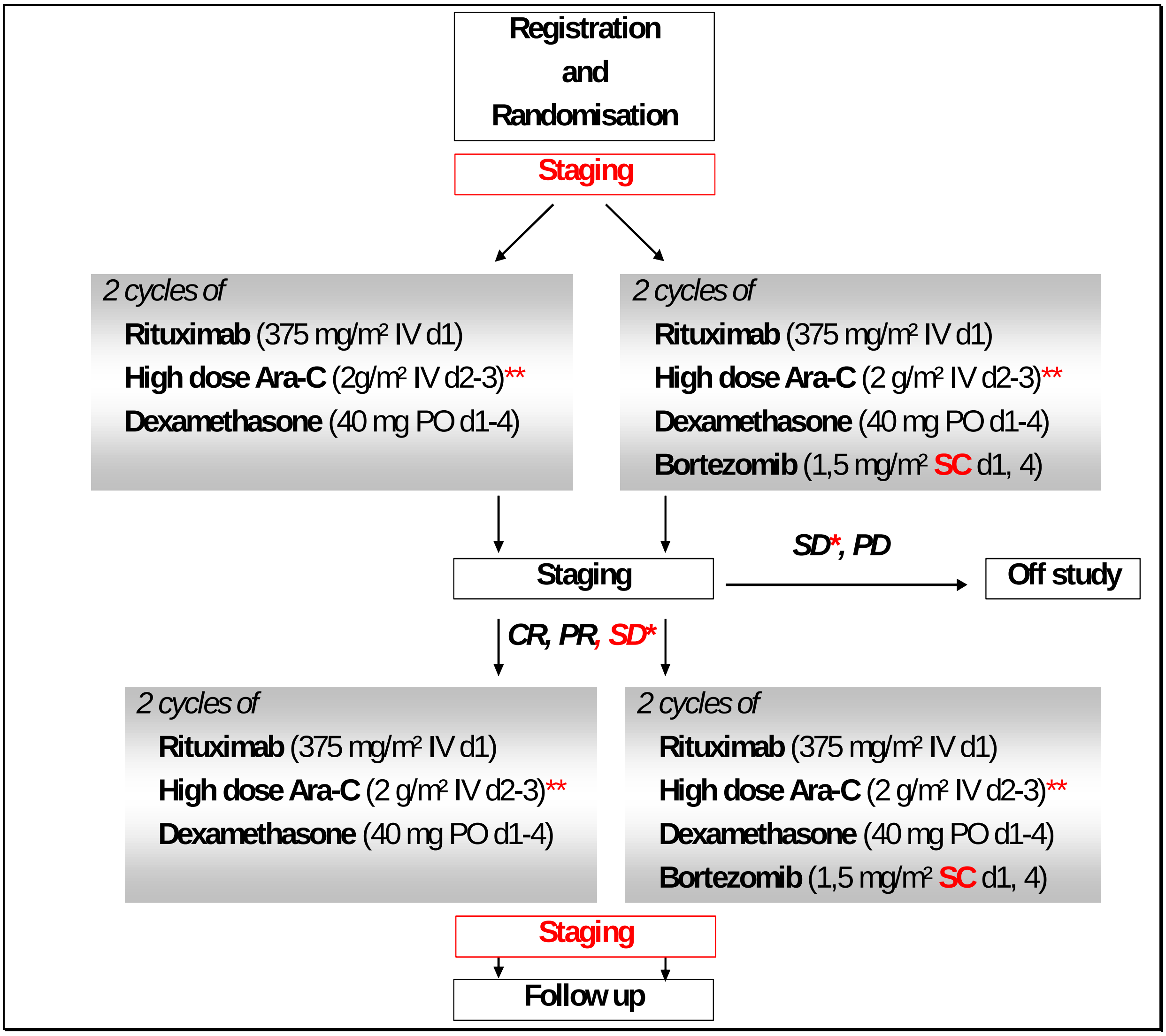

* In case of stable disease, patients may proceed with the treatment at the investigator's discretion.

** *Patients >65 years or s/p myeloablative treatment:* 1000 mg/m²

IV PATHOLOGY REVIEW

One stained (hematoxylin and eosin) slide of a representative lymph node biopsy together with the paraffin embedded block and/or 10 unstained sections on APES-coated smears should be sent to the annotated pathologists below (participants of the European MCL Network Pathology Group). The diagnosis of the local pathologist will be used for registration and start of treatment. However, it is strongly advised – given the high percentage of discordances – to have the material reviewed before entry in the study.
A central pathology review will be performed by the *European MCL Network* Pathology Panel. The review will be blinded with respect to the treatment arm and patient outcome and will comprise the confirmation of the diagnosis of mantle cell lymphoma (both by morphology and immunophenotyping including CD5, CD10, CD20, CD23, BCL2 and Cyclin D1), and recording of the morphological variants including prognostic factors such as Ki67 expression. Finally, for each case freshly frozen material should be available for the design of clone-specific immunoglobulin primers to be used for minimal residual disease (MRD) analysis. If the laboratory has no facility to store these materials, these should be sent to the laboratory of one of the annotated pathologists. If no freshly frozen material is available (for example, if a patient already has had his/her confirmed pathological diagnosis prior to enrollment), this should be stated in the patient's file.

Overview of reference pathologists

Country	**Name and address**
**France**	Dr. Anne MOREAU<br>Service d'Anatomie et Cytologie Pathologiques<br>CHU de Nantes<br>1 pl Alexis Ricordeau<br>44093 Nantes cedex 1<br>Phone: +33-2-40-16-54-48<br>Fax: +33-2-40-16-54-49<br><br>Dr. Danielle CANIONI<br>Service d'Anatomie Pathologique<br>Hôpital Necker-enfants malades<br>75743 Paris<br>Phone: +33-1-44-49-49-92<br>Fax: +33-1-44-49-49-99

**Germany**	Universitätsklinikum Schleswig-Holstein, Campus Lübeck<br>Prof. Dr. A. C. Feller<br>Institut für Pathologie<br>Ratzeburger Allee 160<br>23538 Lübeck<br>Phone: +49-451-500-2707<br>Fax: +49-451-500-3328<br><br>Universitätsklinikum Schleswig-Holstein, Campus Kiel<br>Prof. Dr. W. Klapper<br>Institut für Pathologie<br>Sektion Hämatopathologie und LK-Register<br>Postfach 7154<br>24171 Kiel<br>Phone: +49-431-597-3425<br>Fax: +49-69-431-597-4129<br><br>Universitätsklinikum Frankfurt / M.<br>Prof. Dr. M.L. Hansmann<br>Dr. Senckenbergisches Institut für Pathologie<br>Theodor-Stern-Kai 7<br>60590 Frankfurt<br>Phone: +49-69-6301-5364<br>Fax: +49-69-6301-5241<br><br>Universitätsklinikum Ulm<br>Prof. Dr. P. Möller<br>Institut für Pathologie<br>Robert Koch-Str. 8<br>89081 Ulm<br>Phone: +49-731-500-56321<br>Fax: +49-731-500-3828<br><br>Universitätsklinikum Würzburg<br>Prof. Dr. A. Rosenwald<br>Institut für Pathologie<br>Josef Schneider-Straße 2<br>97080 Würzburg<br>Phone: +49-931-31-81199<br>Fax: +49-931-201-47440<br><br>Pathodiagnostik Berlin<br>Berliner Referenzzentrum für Lymphom- und Hämatopathologie<br>Prof. Dr. H. Stein<br>Komturstr. 58 – 62<br>12099 Berlin<br>Phone: +49-30-2360-842-10<br>Fax: +49-30-2360-842-19
**Italy**	Prof. S. Pileri and M. Milani<br>Istituto di Ematologia, Policlinio & Orsola<br>Via Massarenti 9<br>I-40139 Bologna<br>Phone: +39-51-6364674<br>Fax: +39-51-6363606

Other centers may refer to the reference pathologist of their choice.

Table of contents

SIGNATURE OF SPONSOR, COORDINATING INVESTIGATORS AND RESPONSIBLE STATISTICIAN 3

II SYNOPSIS 4

III TRIAL DESIGN 6

IV PATHOLOGY REVIEW 7

1 BACKGROUND 11

1.1 MANTLE CELL LYMPHOMA 11
1.2 CURRENT TREATMENT OF MCL 11
1.2.1 Conventional chemotherapy and myeloablative treatment 11
1.2.2 Rituximab 11
1.3 INVESTIGATIONAL DRUG (BORTEZOMIB®) 12
1.3.1 Mechanism of action 12
1.3.2 Pharmacology of Bortezomib 12
1.3.3 Potential Adverse Effects of Bortezomib (Velcade®, risk section v. Jan, 2014 13
1.3.4 Clinical trials evaluating bortezomib with special reference to MCL 16
1.3.5 Clinical trials evaluating Bortezomib in combination with chemotherapy 17

2 TRIAL OBJECTIVES 20

3 INVESTIGATIONAL PLAN 20

3.1 OVERALL TRIAL DESIGN 20
3.2 NUMBER OF SUBJECTS AND DURATION OF STUDY 21
3.3 SELECTION OF STUDY POPULATION 21
3.3.1 Inclusion criteria 21
3.3.2 Exclusion criteria 22
3.3.3 Removal of subjects from study treatment 22
3.4 REGISTRATION AND RANDOMIZATION 22
3.5 TREATMENT SCHEDULE 23
3.5.1 Immuno-chemotherapy (R-HAD) 23
3.5.2 Dose Reduction and treatment delay of R-HAD 24
3.5.3 Bortezomib 25
3.5.4 Dose Reduction and treatment delay of Bortezomib 26
3.5.5 Follow-up 27
3.6 SUPPORTIVE CARE 27

4 DIAGNOSTIC PROCEDURES 29

4.1 TABLE OF DIAGNOSTIC TESTS 29
4.2 SAFETY MEASUREMENTS 31
4.3 EVALUATION OF TREATMENT AND RESPONSE 31
4.3.1 Response criteria and Time schedule of Reponse evaluation 31
4.3.2 Baseline evaluations 31
4.3.3 Midterm staging procedures 32
4.3.4 End of treatment evaluation 32
4.3.5 Follow-up evaluations 32
4.3.6 European MCL Research Network/Minimal Residual Disease (MRD) 33

5 TOXICITIES 34

5.1 ADVERSE EVENTS 34
5.2 SERIOUS ADVERSE EVENTS 34
5.3 EXPECTED TREATMENT RELATED SAE 35

6 CAUSES OF DEATH 36

7 STATISTICAL METHODS 37

7.1 STATISTICAL EVALUATION OF THE PRIMARY TRIAL ENDPOINT 37

7.2 NUMBER OF SUBJECTS AND EXPECTED TRIAL DURATION 38
7.3 STATISTICAL METHODS FOR SECONDARY ANALYSES 39
7.4 EVALUATION DURING THE TRIAL 40
7.5 FINAL AND INTERIM REPORTS AND TERMINATION OF THE TRIAL 40

8 ADMINISTRATIVE REQUIREMENTS 41

8.1 GOOD CLINICAL PRACTICE 41
8.2 ETHICAL CONSIDERATIONS 41
8.3 FINANCING AND INSURANCE 41
8.4. PATIENT INFORMATION AND CONSENT 41
8.5. PATIENT CONFIDENTIALITY 41
8.6 CHANGES TO THE PROTOCOL AND PROTOCOL COMPLIANCE 41
8.7 MONITORING 42
8.8 ON SITE AUDITS 42
8.9 DRUG ACCOUNTABILITY 42
8.10 PREMATURE CLOSURE OF THE STUDY 42
8.11 END OF STUDY 42
8.12 RECORD RETENTION 42
8.13 PUBLICATION POLICY 43
8.14 NATIONAL REQUIREMENTS 43

9 REFERENCES 44

10 ABBREVIATIONS 46

11 APPENDIX 47

12.1 WHO PERFORMANCE CRITERIA 47
12.2 ECOG PERFORMANCE CRITERIA 48
12.3 CATEGORIES OF STAGING (ACCORDING TO ANN ARBOR) 49
12.4 RESPONSE CRITERIA 49

1 BACKGROUND

1.1 MANTLE CELL LYMPHOMA

Mantle cell lymphoma (MCL) represents 5–10% of malignant lymphomas and may present with a broad cytological and histological spectrum, which may hamper the diagnosis based on morphology alone. Cytologically two types of MCL have been described: the classical form and its variants (small cell, blastoid and pleomorphic MCL) [1]. Although this subentity of malignant lymphoma had been already described almost two decades ago as centrocytic lymphoma by the KIEL classification it was not before the introduction of the REAL and WHO classifications that MCL has been generally accepted as a distinct entity [1]. The term MCL is derived from the physiological counterpart of the lymphoma cells which is believed to be cells of the mantle zone of lymph node follicles. The cytogenetic hallmark of MCL is the chromosomal translocation t(11;14)(q13;q32) resulting in a constitutive overexpression of the putative oncogene *CCDN1* (*PRAD1, Cyclin D1*) in virtually all cases of MCL [2]. Patients have a median age of >60 years with a predominance of the male sex [3]. The disease presents mostly with advanced Ann Arbor stages (>80% stage IV), bulky tumor mass, generalized lymphadenopathy and involvement of bone marrow, liver or other extranodal manifestation. In 60% of cases massive splenomegaly, hepatomegaly and bulky disease is present at initial diagnosis, but only in less than 50% B-symptoms are found [4].

1.2 CURRENT TREATMENT OF MCL

1.2.1 CONVENTIONAL CHEMOTHERAPY AND MYELOABLATIVE TREATMENT

Despite a rather indolent morphology, conventional chemotherapy is a non-curative approach and does not improve the dismal clinical outcome of MCL with a median survival of 3 years and virtually no long-term survivor [4]. Thus improvement of clinical outcome is urgently warranted.

The introduction of myeloablative radiochemotherapy followed by autologous stem cell transplantation significantly reduced relapse rate in MCL. In an international prospective randomized phase III study the *European MCL Network* has demonstrated a significant prolongation of the progression-free survival after fractionated total body irradiation (12 Gray) plus cyclophosphamide (60mg/kg) followed by autologous stem cell transplantation (ASCT) as compared to standard interferon alpha maintenance in patients with advanced MCL [5]: median PFS was 39 months in the ASCT arm as compared with 17 months for patients in the IFN alpha arm (P = .0108). The 3-year overall survival (OS) was 83% after ASCT versus 77% in the IFN group (P = .18). However, these benefits may be hampered by long term side effects of TBI, namely secondary neoplasias (estimated 5 year risk for t-MDS/ t-AML following PBSCT 3,8% vs. 0% following interferon [6].

Ara-C (cytarabine) has long been proven to be an effective drug in the treatment of many NHL. By combining this agent with cisplatin and dexamethasone the DHAP regimen was introduced by Velasquez et al. in 1988 [7]. Since then many studies have investigated regimens containing high dose Ara-C, especially in the context of anticipated myeloablative therapy in MCL [8, 9]. A French study could demonstrate that a sequential DHAP regimen is very efficient in inducing CR in MCL patients who showed only partial response after 4 cycles of CHOP as first line treatment [10]. Similarly
the addition of Rituximab to the DHAP regimen resulted was feasible and effective in patients with aggressive lymphoma including MCL, who relapsed or were refractory after a CHOP-like regimen (n= 61, ORR 54%) [11].
By adding high- dose Ara-C and Rituximab to initial treatment of MCL patients (122 included, 88 evaluable for response) prior to myeloablative chemotherapy and PBSCT, another study group could demonstrate increased clinical response rate pretransplant, better molecular response rate posttransplant, increased number of tumor cell free grafts and improved failure-free-, relapse-free- and overall-survival (85 % 3 year overall survival as compared to 60 %) [12]. Based on these encouraging results the *European MCL Network* has recently initiated a phase III study comparing alternating courses of CHOP and DHAP in combination with Rituximab with 6 courses of R-CHOP as induction therapy followed by myeloablative consolidation.
Thus high-dose Ara-C represents a standard approach especially in MCL relapsed or refractory after a CHOP like regimen.

1.2.2 RITUXIMAB

In nearly all mantle cell lymphomas a high expression of CD20 may be detected [1, 13]. Rituximab monotherapy has documented only moderate activity in MCL [14-16]. In contrast a combined immunochemotherapy approach has been proven to be superior in 2 randomized GLSG trials. The GLSG has evaluated CHOP alone or in combination with Rituximab in first line therapy of 122 MCL patients. R-CHOP was significantly superior to CHOP in terms of overall response rate (94% vs. 75%; p = 0.0054), complete remission rate (34% vs. 7%; p = 0.00024) and time to treatment failure (TTF; median 21 vs. 14 months; p = 0.0131). No differences were observed for the

progression-free survival. Toxicity was acceptable, with no major differences between the two therapeutic groups [17].
Similarly, in relapsed MCL, the addition of Rituximab to the FCM regimen resulted in a 30% increase of the CR rate and a 20% improve of overall response; interestingly these differences resulted in a significantly improved overall survival in patients with relapsed MCL [18].
Similarly in a historical comparison a study group of the M.D. Anderson Cancer Center could demonstrate encouraging results in MCL patients for the high-dose regimen Hyper-CVAD (fractionated cyclophosphamide, vincristine, doxorubicin and dexamethasone) when combined with Rituximab [19].

Despite these therapeutic improvements the vast majority of patients will eventually relapse. The only potentially curative approach so far is allogeneic bone marrow transplantation [9, 20].

However the majority of MCL patients do not qualify for highly aggressive treatment as the median age at initial diagnosis is above 60 years [13].

1.3 INVESTIGATIONAL DRUG (BORTEZOMIB®)

Bortezomib (Velcade®) has been evaluated in various phase I, II and III trials in both hematologic malignancies and solid tumors, and is currently approved by the US Food and Drug Administration (FDA) and the European Medicines Agency (EMA) for the treatment of multiple myeloma patients who have received at least 2 prior therapies and have demonstrated disease progression on the last therapy.

1.3.1 MECHANISM OF ACTION

Bortezomib (Velcade™), formerly named PS-341, represents a novel and currently unique antineoplastic agent. This small molecule, a modified dipeptidyl boronic acid of only 284 Dalton, is a potent, reversible and specific inhibitor of the 26 S proteasome.

The ubiquitin/ proteasome system degrades most proteins of the cytosol and nucleus and therefore represents a key regulator of cellular protein hemostasis present and abundant in virtually all cells. The 26 S proteasome is composed of two subcomplexes: a barrel-shaped catalytic 20 S core particle and two 19 S regulatory particles capping both ends. Proteins that need to be degraded are “tagged” by a specific and regulated ubiquitination process. Substrates enter the proteasome through a narrow pore, that is largely influenced by the 19 S regulatory particle. Bortezomib inhibits the rate-limiting step of proteolysis by binding to the chymotryptic site of the inner layer of the 20 S core particle [21-25].
Despite detailed knowledge concerning molecular events, the exact mechanism leading to tumor cell death is still unknown. Tumor cells as well as components of the microenvironment seem to be affected. In-vitro and in-vivo studies indicate, that direct induction of apoptosis, inhibition of NF-kappa B, blocking of intra- und extracellular signaling transduction and disturbance of survival pathways seem to contribute to the antineoplastic effect.

1.3.2 PHARMACOLOGY OF BORTEZOMIB

Bortezomib is predominantly inactivated via cytochrome P450 metabolism. Oxidative deboronization accounts for more than 90% of plasma clearance resulting in inactive metabolites in the 20 S proteasome assay.

In solid tumor patients, the mean terminal elimination half life of Bortezomib was 9.06 hours. The mean area under the curve $(AUC)_{(0-24)}$ after the first dose (1.3 mg/m^2) of Bortezomib was 48.2 hr*ng/mL. The average clearance of Bortezomib following a single 1.3 mg/m^2 dose was 49.0 L/hr. After the third dose in the first cycle the AUC increased to 81.0 hr*ng/mL as a result of a reduction in systemic clearance to 28.2 L/hr with a consequent increase in elimination half-life to 54.0 hours. Clinical experience has shown that the change in clearance does not result in overt toxicity from accumulation in this multidose regimen in humans.
In subjects with advanced malignancies, the maximum pharmacodynamic effect (inhibition of 20 S activity) occurred within 1-hour post dose. At the therapeutic dose of 1.3 mg/m^2 in subjects with multiple myeloma, the mean proteasome inhibition at 1-hour post dose was approximately 61%.
The time course of proteasome inhibition in subjects is characterized by maximum inhibition observed within the first hour after administration, followed by partial recovery of proteasome activity over the next 6 to 24 hours to within 50% of the pretreatment activity and almost complete recovery of proteasome activity after 72 hours. Thus a Day 1, 4, 8, and 11 schedule has been established. In theory, this advantage allows cells to recover proteasome activity for normal cellular housekeeping functions between doses.
No formal studies in renal or hepatic dysfunction were done. However in phase II trials, multiple myeloma patients with a creatinine clearance of as low as 13,8 ml/min were dosed with Bortezomib [26].

1.3.3 POTENTIAL ADVERSE EFFECTS OF BORTEZOMIB (VELCADE®, RISK SECTION V. JAN, 2014

The most commonly reported adverse reactions during treatment with VELCADE are nausea, diarrhoea, constipation, vomiting, fatigue, pyrexia, thrombocytopenia, anaemia, neutropenia, peripheral neuropathy (including sensory), headache, paraesthesia, decreased appetite, dyspnoea, rash, herpes zoster and myalgia. (VELCADE SmPC 2014).
Very common side effects of VELCADE (ie ≥1/10) observed in subjects are thrombocytopenia*, neutropenia*, anaemia*, decreased appetite, neuropathies*, peripheral sensory neuropathy, dysaesthesia*, neuralgia*, nausea and vomiting symptoms*, diarrhoea*, constipation, musculoskeletal pain*, pyrexia*, fatigue and asthenia.

Common side effects of VELCADE (ie ≥1/100 to <1/10) observed in subjects are Herpes zoster (incl disseminated and ophthalmic), pneumonia*, Herpes simplex*, fungal infection*, leukopenia*, lymphopenia, dehydration, hypokalaemia*, hyponatraemia*, blood glucose abnormal*, hypocalcaemia*, enzyme abnormality*, mood disorders and disturbances*, anxiety disorder*, sleep disorders and disturbances*, motor neuropathy*, loss of consciousness (inc syncope), dizziness*, disgeusia*, lethargy, headache*, eye swelling*, vision abnormal*, conjunctivitis*, vertigo*, hypotension*, orthostatic hypotension, hypertension*, dyspnoea*, epistaxis, upper/lower respiratory tract infection*, cough*, gastrointestinal haemorrhage (incl mucosal)*, dyspepsia, stomatitis*, abdominal distension, oropharyngeal pain*, abdominal pain (incl gastrointestinal and splenic pain)*, oral disorder*, flatulence, hepatic enzyme abnormality*, rash*, pruritus*, erythema dry skin, muscle spasms*, pain in extremity, muscular weakness, renal impairment*, oedema (incl peripheral), chills, pain*, malaise*, weight decreased.

Uncommon side effects of VELCADE (ie ≥1/1.000 to <1/100) observed in subjects are infection*, bacterial infections*, viral infections*, sepsis (inc septic shock)*, bronchopneumonia, herpes virus infection*, meningoencephalitis herpetic, bacteraemia (inc staphylococcal), hordeolum, influenza, cellulitis, device related infection, skin infection*, ear infection*, staphylococcal infection, tooth infection*, pancytopenia*, febrile neutropenia, coagulopathy*, leukocytosis*, lymphadenopathy, haemolytic anaemia, angioedema, hypersensitivity*, Cushing's syndrome*, hyperthyroidism*, inappropriate antidiuretic hormone secretion, tumour lysis syndrome, failure to thrive*, hypomagnesaemia*, hypophosphataemia*, hyperkalaemia*, hypercalcaemia*, hypernatraemia*, uric acid abnormal*, Diabetes mellitus*, fluid retention, mental disorder*, hallucination*, psychotic disorder*, confusion*, restlessness, tremor, peripheral sensorimotor neuropathy, dyskinesia*, cerebellar coordination and balance disturbances*, memory loss (excl dementia)*, encephalopathy*, posterior reversible encephalopathy syndrome, neurotoxicity, seizure disorders*, post herpetic neuralgia, speech disorder*, restless legs syndrome, migraine, sciatica, disturbance in attention, reflexes abnormal*, parosmia, eye haemorrhage*, eyelid infection*, eye inflammation*, diplopia, dry eye*, eye irritation*, eye pain, lacrimation increased, eye discharge, dysacusis (inc tinnitus)*, hearing impaired (up to and inc deafness), ear discomfort*, cardiac tamponade, cardio-pulmonary arrest*, cardiac fibrillation (inc atrial), cardiac failure (inc left and right ventricular)*, arrhythmia*, tachycardia*, palpitations, Angina pectoris, pericarditis (inc pericardial effusion)*, cardiomyopathy*, ventricular dysfunction*, bradycardia, cerebrovascular accident, deep vein thrombosis*, haemorrhage*, thrombophlebitis (inc superficial), circulatory collapse (inc hypovolaemic shock), phlebitis, flushing*, haematoma (inc perirenal)*, poor peripheral circulation*, vasculitis, hyperaemia (inc ocular)*, pulmonary embolism, pleural effusion, pulmonary oedema (inc acute), pulmonary alveolar haemorrhage, bronchospasm, chronic obstructive pulmonary disease*, hypoxaemia*, respiratory tract congestion*, hypoxia, pleurisy*, hiccups, rhinorrhoea, dysphonia, wheezing, pancreatitis (inc chronic)*, haematemesis, lip swelling*, gastrointestinal obstruction (inc ileus)*, abdominal discomfort, oral ulceration*, enteritis*, gastritis*, gingival bleeding, gastrooesophageal reflux disease*, colitis (inc clostridium difficile)*, colitis ischaemic, gastrointestinal inflammation*, dysphagia, irritable bowel syndrome, gastrointestinal disorder NOS, tongue coated, gastrointestinal motility disorder*, salivary gland disorder*, hepatotoxicity (inc liver disorder), hepatitis*, cholestasis, erythema multiforme, urticaria, acute febrile neutrophilic dermatosis, toxic skin eruption, toxic epidermal necrolysis, Stevens-Johnson syndrome, dermatitis*, hair disorder*, petechiae, ecchymosis, skin lesion, purpura, skin mass*, psoriasis, hyperhidrosis, night sweats, decubitus ulcer, acne*, blister*, pigmentation disorder*, muscle twitching, joint swelling, arthritis*, joint stiffness, myopathies*, sensation of heaviness, renal failure acute, renal failure chronic*, urinary tract infection*, urinary tract signs and symptoms*, haematuria*, urinary retention, micturition disorder*, proteinuria, azotaemia, oliguria*, pollakiuria, vaginal haemorrhage, genital pain*, erectile dysfunction, general physical health deterioration*, face oedema*, injection site reaction*, mucosal disorder*,chest pain, gait disturbance, feeling cold, extravasation*, catheter related complication *, change in thirst*, chest discomfort, feeling of body temperature change*, injection site pain *, hyperbilirubinaemia*, protein analyses abnormal*, weight increased, blood test abnormal*, C-reactive protein increased, fall, contusion.

Rare side effects of VELCADE (ie ≥1/10.000 to <1/1.000) observed in subjects are meningitis (inc bacterial), Eppstein-Barr-virus infection, genital herpes, tonsillitis, mastoiditis, post viral fatigue syndrome, neoplasm malignant, leukaemia plasmacytic, renal cell carcinoma, mass, mycosis fungoides, neoplasm benign*, disseminated intravascular coagulation, thrombocytosis*, hyperviscosity syndrome, platelet disorder NOS, thrombocytopenic purpura, blood disorder NOS, haemorrhagic diathesis, lymphocytic infiltration, anaphylactic shock, amyloidosis, type III immune complex mediated reaction, hypothyroidism, hypermagnesaemia*, acidosis, electrolyte imbalance*, fluid overload, hypochloraemia*, hypovolaemia, hyperchloraemia*, hyperphosphataemia*, metabolic disorder, Vitamin B complex deficiency, Vitamin B12 deficiency, gout, increased appetite, alcohol

intolerance, suicidal ideation*, adjustment disorder, delirium, libido decreased, cerebral haemorrhage*, haemorrhage intracranial (inc subarachnoid)*, brain oedema, transient ischaemic attack, coma, autonomic nervous system imbalance, autonomic neuropathy, cranial palsy*, paralysis*, paresis*, presyncope, brain stem syndrome, cerebrovascular disorder, nerve root lesion, psychomotor hyperactivity, spinal cord compression, cognitive disorder NOS, motor dysfunction, nervous system disorder NOS, radiculitis, drooling, hypotonia, corneal lesion*, exophthalmos, retinitis, scotoma, eye disorder (inc eyelid) NOS,, dacryoadenitis acquired, photophobia, photopsia, optic neuropathy, different degrees of visual impairment (up to blindness)*, ear haemorrhage, vestibular neuronitis, ear disorder NOS, atrial flutter, myocardial infarction*, atrioventricular block*, cardiovascular disorder (inc cardiogenic shock), torsade de pointes, angina unstable, cardiac valve disorders*, coronary artery insufficiency, sinus arrest, peripheral embolism, lymphedema, pallor, erythromelalgia, vasodilatation, vein discolouration, venous insufficiency, respiratory failure, acute respiratory distress syndrome, apnoea, pneumothorax, atelectasis, pulmonary hypertension, haemoptysis, hyperventilation, orthopnoea, pneumonitis, respiratory alkalosis, tachypnoea, pulmonary fibrosis, bronchial disorder*, hypocapnia*, interstitial lung disease, lung infiltration, throat thightness, dry throat, increased upper airway secretion, throat irritation, upper-airway cough syndrome, pancreatitis acute, peritonitis*, tongue oedema*, ascites, oesophagitis, cheilitis, faecal incontinence, anal sphincter atony, faecaloma*, gastrointestinal ulceration and perforation*, gingival hypertrophy, megacolon, rectal discharge, oropharyngeal blistering*, lip pain, periodontitis, anal fissure, change of bowel habit, proctalgia, abnormal faeces, hepatic failure, hepatomegaly, Budd-Chiari syndrome, cytomegalovirus hepatitis, hepatic haemorrhage, cholelothiasis, skin reaction, Jessner's lymphocytic infiltration, Palmar-plantar erythrodysaesthesia syndrome, haemorrhage subcutaneous, livedo reticularis, skin induration, papule, photosensitivity reaction, seborrhoea, cold sweat, skin disorder NOS, erythrosis, skin ulcer, nail disorder, rhabdomyolysis, temporomandibular joint syndrome, fistula, joint effusion, pain in jaw, bone disorder, musculoskeletal and connective tissue infections and inflammations*, synovial cyst, bladder irritation, testicular disorder, prostatitis, breast disorder female, epididymal tenderness, epididymitis, pelvic, pain, vulval ulceration, aplasia, gastrointestinal malformation, ichthyosis, death (inc sudden), multi-organ failure, injection site haemorrhage*, hernia (inc hiatus)*, impaired healing*, inflammation, injection site phlebitits*, tenderness, ulcer, irritability, non-cardiac chest pain, catheter site pain, sensation of foreign body, blood gases abnormal*, electrocardiogram abnormalitits (inc QT prolongation)*, international normalized ratio abnormal*, gastric pH decreased, platelet aggregation increased, troponin I increased, virus identification and serology*, urine analysis abnormal*, transfusion reaction, fractures*, rigors*, face injury, joint injury*, burns, laceration, procedural pain, radiation injuries*, macrophage activation.

Complications arising from these Velcade® toxicities may result in death.

Special precautions for use:

- Gastrointestinal toxicity
  Gastrointestinal toxicity, including nausea, diarrhea, vomiting and constipation are very common with VELCADE treatment. Cases of ileus have been uncommonly reported. Therefore, patients who experience constipation should be closely monitored.
- Haematological toxicity
  VELCADE treatment is very commonly associated with haematological toxicities (thrombocytopenia, neutropenia and anaemia). In the Phase III study evaluating VELCADE (injected intravenously) versus dexamethasone, the most common haematologic toxicity was transient thrombocytopenia. In a Phase II study, platelets were lowest at day 11 of each cycle of VELCADE treatment. There was no evidence of cumulative thrombocytopenia, including in the Phase II extension study. The mean platelet count nadir measured was approximately 40% of baseline. In patients with advanced myeloma the severity of thrombocytopenia was related to pre-treatment platelet count: for baseline platelet count < 75.000/µl, 90% of 21 patients had a count ≤ 25.000/µl during the study, including 14% < 10.000/µl; in contrast, with a baseline platelet count > 75.000/µl, only 14% of 309 patients had a count ≤ 25 x 10^9/l during the study. Platelet counts should be monitored prior to each dose of VELCADE. VELCADE therapy should be withheld when the platelet count is < 25.000/µl or in combination with melphalan and prednisone when the platelet count is ≤ 30.000/µl and re-initiated at a reduced dose after resolution. Potential benefit of the treatment should be carefully weighed against the risks, particularly in case of moderate to severe thrombocytopenia and risk factors for bleeding.
  Therefore, complete blood counts (CBC) with differential and including platelet counts should be frequently monitored throughout treatment with VELCADE.
- Herpes zoster virus reactivation
  Antiviral prophylaxis should be considered in patients being treated with VELCADE. In the Phase III study in patients with previously untreated multiple myeloma, the overall incidence of herpes zoster reactivation was more common in patients treated with VELCADE + Melphalan + Prednisone (14% versus 4% respectively).
- Progressive multifocal leukoencephalopathy (PML)
  Very rare cases with unknown causality of John Cunningham (JC) virus infection, resulting in PML and death, have been reported in patients treated with VELCADE. Patients diagnosed with PML had prior of concurrent immunosuppressive therapy. Most cases of PML were diagnosed within 12 months of their first dose of VELCADE. Patients should be monitored at regular intervals for any new or worsening neurological symptoms or signs that may be suggestive of PML as part of the differential diagnosis of CNS problems. If a diagnosis of PML is suspected, patients should be referred to a specialist in PML and appropriate diagnostic measures for PML should be initiated. Discontinue VELCADE if PML is diagnosed.

- Peripheral neuropathy
  Treatment with VELCADE is very commonly associated with peripheral neuropathy, which is predominantly sensory. However, cases of severe mototr neuropathy with or without sensory peripheral neuropathy have been reported. The incidence of peripheral neuropathy increases early in the treatment and has been observed to peak during cycle 5.
  It is recommended that patients be carefully monitored for symptoms of neuropathy such as a burning sensation, hyperesthesia, hypoesthesia, paraesthesia, discomfort, neuropathic pain or weakness.
  In the Pase III study comparing VELCADE administered intravenously versus subcutaneously, the incidence of Grade ≥ 2 peripheral neuropathy events was 24% for the subcutaneous injection group versus 41% for the intravenous injection group (p=0,0124). Grade ≤ 3 peripheral neuropathy occurred in 6% of patients in the subcutaneous group, compared with 16% in the intravenous treatment group (p= 0,0264). The incidence of all grade peripheral neuropathy with VELCADE administered intravenously was lower in historical studies with VELCADE administered intravenously than in study MMY-3021.
  Patients experiencing new or worsening peripheral neuropathy should undergo neurological evaluation and may require a change in the dose, schedule or route of administration to subcutaneous. Neuropathy has been managed with supportive care and other therapies.
  Early and regular monitoring for symptoms of treatment-emergent neuropathy with neurological evaluation should be considered in patients receiving VELCADE in combination with medicinal products known to be associated withg neuropathy (e.g. thalidomide) and appropriate dose reduction or treatment discontinuation should be considered.
  In addition to peripheral neuropathy, there may be a contribution of autonomic neuropathy to some adverse reactions such as postural hypotension and severe constipation with ileus. Information on autonomic neuropathy and its contribution to these undesirable effects is limited.
- Seizures
  Seizures have been uncommonly reported in patients without previous history of seizures or epilepsy. Special care is required when treating patients with any risk factors for seizures.
- Hypotension
  VELCADE treatment is commonly associated with orthostatic/postural hypotension. Most advers reactions are mild to moderate in nature and are observed throughout treatment. Patients who developed orthostatic hypotension on VELCADE (injected intravenously) did not have evidence of orthostatic hypotension prior to treatment with VELCADE. Most patients required treatment for their orthostatic hypotension. A minority of patients with orthostatic hypotension experienced syncopal events. Orthostatic/postural hypotension was not acutely related to bolus infusion with VELCADE. The mechanism of this event is unknown although a component may be due to autonomic neuropathy. Autonomic neuropathy may be related to bortezomib or bortezomib may aggravate an underlying condition such as diabetic or amyloidotic neuropathy. Caution is advised when treating patients with a history of syncope receiving medicinal products known to be associated with hypotension; or who are dehydrated due to recurrent diarrhea or vomiting. Management of orthostatic/postural hypotension may include adjustment of antihypertensive medicinal products, rehydration or administration of mineralocorticosteroids and/or sympathomimetics. Patients should be instructed to seek medicinal advice if they experience symptoms of dizziness, light-headedness or fainting spells.
- Posterior Reversible Encephalopathy Sandrome (PRES)
  There have been reports of PRES in patients receiving VELCADE. PRES is a rare, often reversible, rapidly evolving neurological condition, which can be present with seizure, hypertension, headache, lethargy, confusion, blindness, and other visual and neurological disturbances. Brain imaging, preferably Magnetic Resonance Imaging (MRI), is used to confirm the diagnosis. In patients developing PRES, VELCADE should be discontinued.
- Heart failure
  Acute development or exaberation of congestive heart failure, and/or new onset of decreased left ventricular ejection fraction has been reported during bortezomib treatment. Fluid retention may be a predisposing factor for signs and symptoms of heart failure. Patients with risk factors for or existing heart disease should be closely monitored.
- Electrocardiogram investigations
  There have been isolated cases of QT-interval prolongation in clinical studies, causality has not been established.
- Pulmonary disorders
  There have been rare reports of acute diffuse infiltrative pulmonary disease of unknown aetiology such as pneumonitis, interstitial pneumonia, lung infiltration, and acute respiratory distress syndrome (ARDS) in patients receiving VELCADE. Some of these events have been fatal. A pre-treatment chst radiograph is recommended to serve as a baseline for potential post-treatment pulmonary changes.
  In the event of worsening pulmonary symptoms (e.g. cough, dyspnea), a prompt diagnostic evaluation should be performed and patients treated appropriately. The benefit/risk ratio should be considered prior to continuing VELCADE therapy.
  In a clinical trial, two patients (out of 2) given high-dose cytarabine (2 g/m^2 per day) by continuous infusion over 24 hours with daunorubicin and VELCADE for relapsed acute mylogenous leukaemia died of ARDS early in the course of therapy, and the study was terminated. Therefore, this specific regimen with concomitant administration with high-dose cytarabine (2 g/m^2 per day) by continuous infusion over 24 hours is not recommended.

- Renal impairment
  Renal complications are frequent in patients with multiple myeloma. Patients with renal impairment should be monitored closely.
- Hepatic impairment
  Rare cases of hepatic failure have been reported in patients receiving VELCADE and concomitant medicinal products and with serious underlying conditions. Other reported hepatic reactions include increases in liver enzymes, hyperbilirubinaemia, and hepatitis. Such changes may be reversible upon discontinuation of bortezomib.
- Tumor lysis syndrome
  Because bortezomib is a cytotoxic agent and can rapidly kill malignant plasma cells, the complications of tumor lysis syndrome may occour. The patients at risk of tumor lysis syndrome are those with high tumor burden prior to treatment. These patients should be monitored closely and appropriate precautions taken.
- Concomitant medicinal products
  Patients should be closely monitored when given bortezomib in combination with potent CYP3A4-inhibitors. Caution should be exercised when bortezomib is combined with CYP3A4- or CYP2C19 substrates.
  Normal liver function should be confirmed and caution should be exercised in patients receiving oral hypoglycemic.
- Potential immunocomplex-mediated reactions
  Potentially immunocomplex-mediated reactions, such as serum-sickness-type reaction, polyarthritis with rash and proliferative glomerulonephritis have been reported uncommonly. Bortezomib should be discontinued if serious reactions occur.

Developmental toxicity studies in the rat and rabbit have shown embryo-fetal lethality at maternally toxic doses, but no direct embryo-foetal toxicity below maternally toxic doses. Fertility studies were not performed but evaluation of reproductive tissues has been performed in the general toxicity studies. In the 6-month rat study, degenerative effects in both the testes and the ovary have been observed. It is, therefore, likely that bortezomib could have a potential effect on either male or female fertility. Peri- and postnatal development studies were not conducted.

Further details on the potential risks of VELCADE may be found in the Summary of Product Characteristics (SmPC).

1.3.4 CLINICAL TRIALS EVALUATING BORTEZOMIB WITH SPECIAL REFERENCE TO MCL

A phase I trial to determined the MTD and dose-limiting toxicity (DLT) in a number of therapeutic settings involving subjects with various advanced malignancies. In the 3-week schedule of Bortezomib monotherapy (4 doses, given on Days 1, 4, 8, and 11 of a 21-day treatment cycle), the DLT occurred at 1.56 mg/m^2/dose (3 subjects with Grade 3 diarrhea and 1 with peripheral sensory neuropathy). Therefore, the MTD at this schedule was at least 4 x 1.3- 1.5 mg/m^2/dose [27-29].

Preliminary clinical data from phase I and phase II studies indicate that Bortezomib has antitumor activity in patients with MCL.

Three MCL subjects were treated in phase I studies of Bortezomib, and 1 experienced a PR [28] In ongoing phase 2 studies of single-agent Bortezomib in previously treated NHL subjects, 19 MCL subjects have been treated [30, 31]. Among these 19 patients, 3 have experienced CR and 6 PR.

In another phase II trial 24 assessable patients with NHL were treated with Bortezomib as a single agent, including 11 cases of MCL. Median number of prior therapies was 2. Bortezomib was dosed 1.5 mg/m² and given as slow intravenous push on days 1, 4, 8 and 11. A median number of 2,5 cycles was applied. Bortezomib was tolerated well with no grade 4 toxicity being observed. Grade 3 toxicity included thrombocytopenia (8%), lymphopenia (21%), sensory neuropathy (4%) and motor neuropathy (4%). Of the 9 evaluable MCL patients 1 CRu and 4 PR were achieved, with time of response ranging from 1 to 19 months. Another 4 MCL patients achieved a SD [32]).

In another phase II trial of Bortezomib (1.5 mg/m² IV push d 1, 4, 8, 11) in 60 patients with relapsed or refractory indolent or aggressive NHL, 33 subjects with MCL were included, 29 of whom being evaluable concerning response. ORR was 41% (6/29 CR, 6/29 PR). Serious adverse were uncommon with grade 3 thrombocytopenia (47 %), GI toxicity (20 %), fatigue (20 %) and neuropathy being most frequent; grade 4 toxicity occurred in 9 patients (15 %) [33].

In a phase II trial 14 patients with stage IV MCL were treated with Bortezomib (1.3 mg/m² IV push d 1. 4, 8, 11). Median number of cycles applied was 4 (range 1- 7). 12 patients were evaluable concerning response with 4 PR, 5 SD and 3 PD being observed [34].

The promising results justify the evaluation of a combination therapy with Bortezomib.

In order to augment chemosensitivity and to overcome antiapoptotic escape mechanisms of tumor cells in response to chemotherapy, several clinical trials are currently evaluating combination therapy with inhibition of proteasome activity.

Based on preclinical data, showing that combination therapy of Bortezomib with many cytotoxic agents proved to be more potent at inducing antitumor activity than either drug alone, several phase II trial are currently evaluating efficacy of Bortezomib plus chemotherapy in solid tumor patients as well as in hematologic neoplasms.

In a phase I study 42 patients with advanced hematologic malignancies were treated with Bortezomib (dose escalation from 0.9 to 1.5 mg/m² on days 1, 4, 8 and 11) combined with pegylated liposomal doxorubicin, PegLD (30 mg/m² on day 4). The MTD based on cycle 1 was 1.5 mg/m² and 30 mg/m² for Bortzomib and PegLD, respectively. Therapy was generally well tolerated with 8 of 22 evaluable patients with multiple myeloma achieving a CR of near-CR, 8 patients a PR [35].

In a phase I/II study 33 patients with relapsed or refractory diffuse large B cell lymphoma (DLBCL) were treated with dose adjusted EPOCH (etoposide, cyclophosphamide, doxorubicin) combined with dose escalated Bortezomib (0.5 to 1.7 mg/m² day 1 and 4). Dose limiting toxicity was autonomic neuropathy in 3 patients treated at a Bortezomib dose of 1.7 mg/m². Therefore MTD for Bortezomib in combination with an anthracycline-based chemotherapy was 1.5 mg/m² [36]

Furthermore a phase II study is currently recruiting patients with relapsed or refractory indolent B-cell lymphoma to a combination therapy consisting of Rituximab and Bortezomib (Trehu E., personal communication). In vitro data suggest synergistic efficacy of Ara-C and Bortezomib in MCL [37, 38].

Thus, based on this encouraging results and developments this randomized phase II trial will compare a combination of Rituximab and high dose Ara-C with or without Bortezomib in patients with relapsed MCL after/ not eligible for myeloablative treatment. Given the efficacy and the favorable safety profile of Rituximab in MCL as discussed in chapter 1.2.3 application of Rituximab has been considered standard treatment and will applied in each arm of the study at a dose of 375 mg/m² intravenously (max. dose 750 mg). To avoid the cumulative myelotoxicity of Bortezomib and high dose Ara-C treatment, Bortezomib will be given only on days 1 and 4 at a dose of 1.5 mg/m² intravenously (Orlowski et al.), resulting in a time-dependent separation of cell nadirs due to proteasome inhibition and chemotherapy.

1.3.5 CLINICAL TRIALS EVALUATING BORTEZOMIB IN COMBINATION WITH CHEMOTHERAPY

In multiple myeloma, numerous trials have evaluated combinations of Bortezomib with various chemotherapy regimens. Based on initial phase I data and a subsequent randomized phase III trial, e.g. liposomal anthracycline in combination with Bortezomib has been meanwhile registered in multiple myeloma (Orlowski et al 2007).

Similarly, an increasing number of phase I/II trials investigating Bortezomib and different chemotherapy regimens in combination have been performed in non-Hodgkin's lymphoma including MCL indicating the feasibility of this approach.

Bortezomib in combination with immuno-chemotherapy

**author**	**Phase**	**disease entity (no. of patients)**	**regimen**	**outcome**
Leonard et al, 2005 [39]	I/II	DLCL (16), MCL (4)	R-CHOP21+ Bortezomib	ORR 95%, CR/CRu 80%. conclusion: combination with Bortezomib 1,3 mg/m² (d1,4) feasable
Dunleavy et al 2005 [36]	I/II	relapsed/ refractory DLCL (33)	DA-EPOCH + Bortezomib	DLT Bortezomib 1,7 mg/m² (d1,4): autonomous neuropathy
Mounier et al 2007 [40]	I/II	FL (18), DLCL (9), MCL (4), MZL (10), LPL (4), SLL (4)	R-CHOP + Bortezomib	CR rate 83%. high neurotoxicity Conclusion: no combination with vincristine recommended
Gerecitano et al 2006 [41]	I/II	relapsed/ refractory indolent lymphoma (16)	R-CP + Bortezomib	PR in 2/9 patients. Dose escalation up to Bortezomib 1,8 mg/m² (d1,8) without DLT
Romaguera 2008 (personal	I/II	relapsed/ refractory MCL (7)	R-Hyper-CVAD	Bortezomib 0.7 mg/m² without DLT

communication)				

In detail, the CHOP regimen was combined with Bortezomib at various doses. Combinations with doses up to 1.5 mg/m2, day 1 and 4 were well tolerated (Leonard et al 2005 [39]), whereas at higher doses (Dunleavy et al 2005 [36]) or continuous schedules (Mounier et al 2007 [40]), a significant neurotoxicity was observed. Therefore, some authors discourage a combination with vinca alkaloids. Thus, in the current trial, a stringent dose reduction should be performed in any case of neurotoxicity according to chapter 3.5.4.

Subsequently, an appropriately modified regimen without vincristine was well tolerated with escalating doses of Bortezomib up to 1,8 mg/m² (d1, 8) without any observed DLT (Gerecitano et al 2006 [41]).

Regimens containing high dose Cytarabine have been used for more than 20 years in the treatment of refractory non-Hodgkin's lymphomas. More specifically, a series of MCL patients treated with high dose Cytarabine have been presented: The MD Anderson has activated a phase I/II study combining Bortezomib with the dose-intensified Hyper-CVAD regimen. With 7 patients included, no DLT was observed so far at a Bortezomib dose of 0.7 mg/m².

Similarly, a total of 8 patients were individually treated with a combination of high dose Cytarabine, dexamethasone and Bortezomib +/- Rituximab (dose and regimen identical to the current phase III trial). In line with the observed *in vitro* synergism (Weigert et al. 2006, 2007 [42, 43]), this combination achieved a disease control in 6 out of these 8 desperate patients with one patient still in ongoing remission after a follow-up of more than 24 months.

Patient characteristics of individually treated patients with relapsed MCL

Evaluable patients	8
Median age	65 years (range 54-76)
Age > 60 years	6/8
Male gender	5/8
Stage III/IV	8/8
BM involvement	3/8
Median no. of prior lines of systemic therapy	
2	2
3	1
4	2
5	1
7	2
Prior rituximab	8/8
Prior CHOP	8/8
Prior HD cytarabine	0/8
Prior bortezomib	2/8
Prior ASCT	1/8

Efficacy of high dose Cytarabine, dexamethasone and Bortezomib +/- Rituximab

Evaluable patients	8
Median time since start of treatment	265 days
No. of applied cycles	24
Patients with 2 cycles	4
Patients with 4 cycles	4
Best response after 2 cycles (n=8)	
CR	0/8
PR	4/8
MR	1/8
SD	1/8
PD	2/8
Best response after 4 cycles (n=4)	
CR	1/4
PR	3/4
Event (progression/relapse/death)	6/8
Time to event	49, 51, 119, 130, 147, 271 days
Salvage therapy	4/8
Ongoing remission	2/8
Time in remission	233+ (PR), 311+ (SD)
Alive	5/8
Death	3/8<br>2 deaths: 75 and 314 days after start of treatment

(both PD after 2 cycles)
1 death: 271 days after start of treatment (in PR after 4 cycles) after allogeneic stem cell transplantation from HLA identical sibling

Even more importantly, the observed hematotoxicity of this regimen was significant, but feasible with only minor differences in comparison to the current first line trial of the *European MCL Network* evaluating a similar regimen (R-DHAP: High-dose Ara-C, dexamethasone and cis-platinum) in younger MCL patients.

Toxicity of R-HAD and Bortezomib in relapsed MCL

Evaluable patients	8
Max. leukopenia (grade 3 / 4)	8/8
Grade 3	2/8
Grade 4	6/8
Max. thrombocytopenia (grade 3 / 4)	7/8
Grade 3	2/8
Grade 4	5/8
Max. anemia (grade 3 / 4)	0/8
Grade 2	6/8
Neutropenic fever (≥grade 2)	2/8
Grade 3	2/8
Infection other than neutropenic fever	2/8
Grade 2	2/8 (herpes zoster)
Max. neuropathy (≥grade 2)	1/8
Grade 3	1/8
Max. other toxicity (≥grade 2) except alopecia	7/8
Grade 2	6/8 (fatigue)
Grade 3	1/8 (hepatotoxicity)
Dose reduction cytarabine	8/8
Dose reduction bortezomib	2/8

Toxicity of R-DHAP in first line treatment of younger MCL patients (Dreyling et al 2007 [44])

Toxicity	Grade	freq	%	Toxicity	Grade	freq	%
Hemoglobin	1 or 2	47	56	Arrhythmia	1 or 2	2	3
	3 or 4	33	39		3 or 4	1	1
Leukocytes	1 or 2	14	16	Cardiac Function	1 or 2	2	3
	3 or 4	63	74		3 or 4	0	0
Granulocytes	1 or 2	7	9	Pulmonary Function	1 or 2	4	5
	3 or 4	60	78		3 or 4	1	1
Platelets	1 or 2	13	15	Hematuria	1 or 2	3	4
	3 or 4	63	75		3 or 4	0	0
Lymphocytes	1 or 2	11	14	Neuropathy	1 or 2	22	27
	3 or 4	58	73		3 or 4	1	1
Creatinine	1 or 2	34	41	Depression	1 or 2	9	11
	3 or 4	3	4		3 or 4	1	1
Bilirubin	1 or 2	6	7	Allergy	1 or 2	4	5
	3 or 4	1	1		3 or 4	0	0
Transaminases	1 or 2	26	33	Weight loss	1 or 2	10	12
	3 or 4	2	3		3 or 4	0	0
Nausea	1 or 2	48	57	Bleeding	1 or 2	2	3
	3 or 4	5	6		3 or 4	1	1
Vomiting	1 or 2	28	34	Alopecia	1 or 2	23	32
	3 or 4	4	5		3 or 4	22	30
Diarrhea	1 or 2	17	21	Fatigue	1 or 2	47	57
	3 or 4	1	1		3 or 4	1	1
Constipation	1 or 2	16	20	Infections	1 or 2	17	21
	3 or 4	0	0		3 or 4	7	9
Mucositis	1 or 2	19	23	Myalgia/Arthralgia	1 or 2	11	14

Toxicity	Grade	freq	%	Toxicity	Grade	freq	%
	3 or 4	1	1		3 or 4	1	1
				Febrile neutropenia	1 or 2	0	0
					3 or 4	16	20

2 TRIAL OBJECTIVES

The objective of this trial is to compare the efficacy and safety of the combination of Rituximab, high-dose Ara-C and dexamethasone (R-HAD) with Bortezomib to R-HAD alone in patients with relapsed or refractory mantle cell lymphoma after or not eligible for myeloablative treatment. The primary trial endpoint is the time to treatment failure (TTF). Study arms will be compared to each other to evaluate the effect of additional Bortezomib. Each study arm will also be compared to historical controls of relapsed MCL (GLSG data [18]).

3 INVESTIGATIONAL PLAN

3.1 OVERALL TRIAL DESIGN

This study is a prospective, randomized, multicenter, open-label phase III clinical trial to compare the efficacy and safety of Bortezomib in combination with Rituximab, high-dose Ara-C and dexamethasone (R-HAD) to R-HAD alone in patients with relapsed or refractory MCL after or not eligible for myeloablative treatment. The primary endpoint is time to treatment failure (TTF). Secondary endpoints are the complete response (CR) rate, the overall response (CR,PR) rate, the progression-free survival (PFS), the progression free survival of responders, the time to next lymphoma treatment, overall survival (OS), safety and tolerability of Rituximab, high-dose Ara-C and dexamethasone alone or in combination with Bortezomib. Study arms will be compared to each other to evaluate the impact of additional Bortezomib. Study arms will also be compared to historical controls (GLSG data [18]).

To be enrolled into the trial, patients must fulfill all inclusion criteria, must not meet any of the exclusion criteria and written informed consent has to be obtained. Baseline evaluations (details in chapter 4.3.2) consist of medical history (with special respect to prior antineoplastic therapy including best response status, duration of response and residual toxicity), documentation of demographic data, complete physical examination (including vital signs, body height and weight, ECOG/WHO performance status and neurologic evaluation), ECG, echocardiography, CT imaging of neck, chest, abdomen and all other lymphoma manifestations, laboratory work-up as listed below and immunophenotyping, bone marrow aspiration and biopsy and additional tissue diagnosis (e.g. lymph node biopsy) if not performed during 6 months prior to study entry.

Randomization is performed centrally, blocked and stratified according to response to initial therapy, International Prognostic Index (IPI) risk factors, previous stem cell transplantation, high dose Ara-C therapy and the respective study group. Randomization is balanced (1:1) between the treatment arms.

Treatment (details in chapter 3.5.1) consists of Rituximab 375 mg/m² (max. dose 750 mg) given intravenously on day 1, followed by Ara-C 2 g/m² given intravenously over a 3-hour period on two consecutive days (day 2 and 3). In patients >65 years of age at time of study entry or post myeloablative treatment, a dose reduction of Ara-C to 1 g/m² will be performed. If patients were randomized to receive combination therapy, Bortezomib 1.5 mg/m² will be given additionally subcutaneous (at least one hour prior to Rituximab infusion) on day 1 and on day 4. Dexamethasone at dose of 40 mg will be given p.o. on four consecutive days (days 1- 4). Treatment course will be repeated in 3-week intervals (day 22 +/- 3 days).

Agent	*Dose*	*Day*	*Route*
Rituximab	375 mg/m²	1	IV
Ara-C	2000 mg/m² (*Patients >65 years or s/p myeloablative treatment:* 1000 mg/m²)	2 and 3	IV (over 3 hrs)
Dexamethasone	40 mg	1 to 4	PO
±Bortezomib	1.5 mg/m²	1 and 4	SC
Treatment course will be repeated in 3-week intervals (day 22 +/- 3 days)			

Safety evaluation (details in chapter 0) will be done throughout the study (treatment and follow-up period) at each visit consisting of medical history, physical examination (including vital signs, weight, performance status) and laboratory work-up as listed below.

Midterm staging procedures (details in chapter 4.3.3) will be performed after 2 treatment cycles (immediately prior to next scheduled treatment cycle). Mandatory staging procedures will consist of medical history, physical examination (including vital signs, weight, performance status), CT evaluation of all initial lymphoma manifestations, ECG, echocardiography and laboratory work-up as listed below. In patients with bone marrow infiltration only, bone marrow aspiration/ biopsy will be considered a mandatory staging procedure. Additional procedures should be performed as clinically indicated judged by the treating physician.
In case of progressive disease patients will be off study. In case of stable disease, patients may proceed with the treatment at the investigator's discretion.
Patients responding to study treatment (in terms of partial or complete response) with acceptable toxicity profile will receive two additional treatment cycles.

Treatment can be stopped at any time during treatment as decided by the patient or the investigator. However all subjects who permanently discontinue treatment have to complete the end of treatment evaluation. The reason(s) for discontinuation have to be recorded accordingly in the subject's case report form (CRF). Subjects withdrawn from the study will not be replaced. Subjects who are withdrawn for any reason may not re-enter this study at any time.

End of treatment evaluation (details in 4.3.4) will be performed 4 weeks after day 1 of last treatment course and consists of medical history, physical examination (including vital signs, weight, performance status), CT imaging of neck, chest, abdomen and all initial lymphoma manifestations, ECG, echocardiography and laboratory work-up as listed below. Bone marrow aspiration/ biopsy will be done if bone marrow was initially infiltrated by lymphoma. Additional evaluation procedures can be performed as clinically indicated by the treating physician.

The mandatory follow up period (details in 4.3.5) of each patient will be 36 months after the patient completed the study treatment. However each individual should be further followed to collect additional data on time to progression and overall survival. Evaluation will be performed in 3 month intervals during the first two years, and in 6 month intervals thereafter consisting of medical history, physical examination (including vital signs, weight, performance status), CT of neck, chest, abdomen and all initial lymphoma manifestations and laboratory work-up as listed below.

3.2 NUMBER OF SUBJECTS AND DURATION OF STUDY

Under the assumption of a 55% hazard reduction for TTF by the addition of Bortezomib to R-HAD, a sample size of approximately 175 subjects and a trial duration of approximately 3.5 years is estimated. The maximum number of events is set to 160 yielding a maximal trial duration of approximately 5.5 years (for further details refer to chapter 8).

3.3 SELECTION OF STUDY POPULATION

3.3.1 INCLUSION CRITERIA

Each subject must fulfill all of the following inclusion criteria before enrollment to the study:

- Confirmed pathological diagnosis of MCL according to WHO classification.
- Relapse or progression following 1 to 3 prior lines of anti-neoplastic standard therapy. Therapy in remission after initial induction like intensified chemotherapy for stem cell separation followed by myeloablative therapy or any kind of maintenance therapy is classified as one line of therapy with the induction therapy.
- If Rituximab was part of prior induction treatment, documented time to progression must be at least 12 weeks after this particular regimen.
- If high-dose Ara-C was part of prior treatment, documented time to progression must be at least 6 months after this particular regimen.
- Patients relapsed after autologous stem cell transplantation or not appropriate for myeloablative treatment.
- At least 1 measurable or assessable site of disease; in case of bone marrow infiltration only, bone marrow aspiration/ biospy is mandatory for all staging evaluations.
- age ≥ 18 years
- ECOG/WHO Performance Score 0-2, unless lymphoma related.
- The following laboratory values at screening, unless lymphoma related:
    - Absolute neutrophil count (ANC) ≥1500 cells/µL
    - Platelets ≥100,000 cells/µL

  - Transaminases (AST and ALT) ≤3 x upper limit of normal (ULN)
  - Total bilirubin ≤2 x ULN
  - Creatinine ≤2 mg/dL or calculated creatinine clearance ≥50 mL/min
- Toxic effects of previous therapy or surgery resolved to NCI CTC grade 2 or better.
- Premenopausal fertile females must agree to use a highly effective method of birth control for the duration of the therapy. A highly effective method of birth control is defined as those which result in a low failure rate (i.e. less than 1% per year) when used consistently and correctly such as implants, injectables, combined oral contraceptives, some IUDs, sexual abstinence or vasectomised partner.
- Men must agree not to father a child for the duration of therapy and must agree to advice a female partner to use a highly effective method of birth control.
- Written informed consent before performance of any study-related procedure.

3.3.2 EXCLUSION CRITERIA

Subjects meeting any of the following exclusion criteria are not to be enrolled in the study:
- Treatment within another clinical trial within 30 days before trial entry or planed during this trial
- Anti-neoplastic (including radiation and antibody treatment) or experimental therapy within 4 weeks before planed Day 1 of Cycle 1 (Nitrosoureas within 6 weeks) or radioimmunoconjugates or toxin immunoconjugates such as Ibritumomab tiuxetan (Zevalin™) or Tositumomab (Bexxar®) within 12 weeks before planed Day 1 of Cycle 1
- Known hypersensitivity to Rituximab, boron or mannitol.
- Active malignancy other than MCL within 5 years before Day 1 of Cycle 1, with the exception of complete resection of basal cell carcinoma, squamous cell carcinoma of the skin, or in situ malignancy.
- Active systemic infection requiring treatment.
- HIV, hepatitis B or C
- Patient has ≥ grade 2 peripheral sensory neuropathy or neuropathic pain defined by the NCI Common Terminology Criteria for Adverse Events (CTCAE).
- Symptomatic degenerative or toxic encephalopathy
- Serious medical condition (such as severe hepatic impairment, pericardial disease, acute diffuse infiltrative pulmonary disease, systemic infections etc) or psychiatric illness likely to interfere with participation in this clinical study.
- Female subject is pregnant or breast-feeding (pregnancy testing is mandatory for premenopausal women).

3.3.3 REMOVAL OF SUBJECTS FROM STUDY TREATMENT

Study treatment is to be permanently discontinued for subjects meeting any of the following criteria:
- Progressive disease
- Unacceptable toxicity
- Concomitant disease, which makes further therapy with (B)R-HAD impossible
- Decision by subject, investigator or study coordinator
- Severe protocol violation
- Death

All subjects who permanently discontinue treatment, whether prematurely or as scheduled, must complete the end of treatment visit and should be followed thereafter.

Subjects will be discontinued from the study for the following reasons:
- Withdrawal of informed consent
- Severe protocol violation
- Loss of follow-up
- Death

The reason(s) for a subject's discontinuation from the study are to be recorded in the source documents and the subject's case report form (CRF).

3.4 REGISTRATION AND RANDOMIZATION

Registration and randomization will be done at the national study group or the central data center in Munich via fax:

European MCL Network data center
Klinikum der Universität München
Dept. of Medicine III
Dr. M. Unterhalt
Marchioninistr. 15
81377 München / GERMANY
Phone: +49-89-4400-74900 -74901
Fax: +49-89-4400-77900 -77901

Eligible patients will be randomized to receive Rituximab, high-dose Ara-C and Dexamethasone alone or in combination with Bortezomib.

Randomization will be stratified according to the following factors:

- Response to initial therapy: relapse vs. primary refractory disease

- International Prognostic Index (IPI) with following risk factors:

  - age > 60 years
  - Ann Arbor stage III and IV
  - LDH serum level over normal range of the respective laboratory
  - WHO/ECOG performance-status >1
  - more than 1 extra nodal involvement

  Stratification-groups: 0 to 2 risk factors (low and low intermediate risk) vs.
  3 to 5 risk factors (high or high intermediate risk)

- previous stem cell transplantation: yes vs. no

- previous therapy with high-dose Ara-C: yes vs. no

- Study group/association of centre:
  - LYSA
  - GLSG
  - Centres not associated to one of these study groups

3.5 TREATMENT SCHEDULE

Study treatment will be administered only to eligible subjects according to inclusion and exclusion criteria after registration and randomization at the data center in Munich.

3.5.1 IMMUNO-CHEMOTHERAPY (R-HAD)

Rituximab 375 mg/m² (max 750 mg) will be given intravenously on day 1. Ara-C 2 g/m² will be given intravenously over a 3-hour on two consecutive days (day 2 and 3). In patients >65 years at time of study entry or post myeloablative treatment, dose reduction of Ara-C to 1 g/m² will be performed. Dexamethasone will be given at a dose of 40 mg p.o. on four consecutive days (days 1 to 4).
Patients randomized to receive combination therapy, Bortezomib 1.5 mg/m² will be given additionally as a subcutaneous injection through the thighs or abdomen (for details please see below) on day 1 (at least one hour prior to Rituximab infusion) and similarly on day 4. Treatment course will be repeated in 3-week intervals (day 22 + / - 3 days).

Subcutaneous injection of Bortezomib:
VELCADE 3.5 mg reconstituted solution is administered subcutaneously through the thighs (right or left) or abdomen (right or left). The solution should be injected subcutaneously, at a 45-90° angle. Injection sites should be rotated for successive injections.

If local injection site reactions occur following VELCADE subcutaneous injection, a switch to intravenous injection is possible.
For dilution information please refer to the SmPC.

Agent	*Dose*	*Day*	*Route*
Rituximab	375 mg/m²	1	IV

Ara-C	2000 mg/m² (*Patients >65 years or s/p myeloablative treatment:* 1000 mg/m²)	2 and 3	IV (over 3 hrs)
Dexamethasone	40 mg	1 to 4	PO
± Bortezomib	1.5 mg/m²	1 and 4	SC
Treatment course will be repeated in 3-week intervals (day 22 +/- 3 days)			

Before each treatment cycle the following laboratory values are required:
- Absolute neutrophil count (ANC) ≥1500 cells/µL
- Platelets ≥100,000 cells/µL
- Creatinine ≤2 mg/dL or calculated creatinine clearance ≥50 mL/min

In case of severe side effects, like severe left ventricular systolic dysfunction treatment has to be withheld and discussed with the study coordinators.

Rituximab 375 mg/m^2 (max 750 mg) will be given only if the number of circulating lymphoma cells is < 20 x 10^9/µl to avoid a cytokine release syndrome more frequently observed in leukemic lymphoma. This criterion has to be reconsidered before each consecutive course.
Dose modifications of Rituximab should not be performed. In case of inacceptable toxicity/ hypersensitivity to Rituximab, application of Rituximab has to be permanently discontinued. However, those patients will be evaluated accordingly (intent to treat).
It is strongly advised to give the first Rituximab infusion in an inpatient setting. If no adverse events have occurred the following infusions can be given in an outpatient ward. A peripheral (IV) line will be established. Vital signs (blood pressure, pulse, respiration, and temperature) should be monitored every 15 minutes during the first hour or until stable and then hourly until the infusion is discontinued and vital signs are stable. Premedication with paracetamol and/or antihistaminics is strongly advised. Dexamethasone 40 mg should be given 1 hour prior to Rituximab (either orally or intravenously). The initial dose of Rituximab should be 50 mg/hr for the first hour. If no adverse event is seen, the infusion rate may be escalated in 30 minutes intervals with increment steps of 50 mg/hr, to a maximum of 400 mg/hr. Patients may experience transient fever and rigors with infusion of chimeric anti-CD20 antibody.
If hypersensitivity or infusion-related events develop, the infusion should be temporarily slowed or interrupted. The patient should be treated according to the appropriate standard of care. The infusion should be continued at half the previous rate after symptoms have abated.

Ara-C is a pyrimidine antagonist and one of the most active agents in the treatment of leukemia and lymphoma.
In this study Ara-C will be given intravenously over a 3-hour period every 24 hours for two consecutive days (day 2 and 3). In patients >65 years at time of study entry or status post myeloablative treatment, there will be a dose reduction of Ara-C to 1 g/m² respectively.
Doses >1.7 g/m² may produce conjunctivitis which can be ameliorated with prophylactic use of corticosteroid eye drops (e.g. Isoptodex™). Dexamethasone eye drops may be administered at 2-3 drops every 6 hours during days 2-4. Observed toxicities associated with high-dose Ara-C therapy include cerebellar toxicity, corneal keratitis, hyperbilirubinemia, pulmonary edema, pericarditis and -tamponade. Other known adverse events are listed below.

3.5.2 DOSE REDUCTION AND TREATMENT DELAY OF R-HAD

No dose modification of Rituximab, high-dose Ara-C and Dexamethasone (R-HAD) will be made in the first course.
During the next courses, modifications of the treatment schedule will be made if myelosuppression occurs: if WBC < 3x 10⁹/l or thrombocytes < 100x 10⁹/l after 3 weeks, postpone up to 2 weeks; if after 5 week insufficient recovery or ongoing non-hematological grade III/IV toxicities (except alopecia), adapt according to scheme below.

WBC x 10⁹/l	thrombocytes x 10⁹/l	Ara-C	Dexamethasone	Rituximab
>3	>100	100%	100%	100%
2-3	75 – 100	75%	100%	100%
1-2	50 – 75	50%	100%	100%
<1	<50	0%	100%	100%

Dose reduction will be calculated according to the doses given in the previous cycle. In case of severe myelosuppression with leucocyte counts <1.0 x 10^9/l (and/or thrombocyte counts < 25 x 10^9/l) as assessed on two consecutive measures but recovery WBC >3.0x 10^9/l (and/or thrombocytes > 100 x 10^9/l) after 3 weeks, it is strongly advised to reduce the dose of R-HAD to 75% in Ara-C in subsequent cycles. This reduction of dose can be omitted if the severe myelosuppression can be assumed to be the result of an initial significant bone marrow involvement.
In case of any ≥ grade 3 non-hematological toxicity despite optimized supportive care (except alopecia and nausea) dose modifications should be discussed with the study coordinators. Reasons have to be recorded in the source documents and the patient's case report form (CRF).

3.5.3 BORTEZOMIB

Bortezomib (Velcade™), formerly named PS-341, represents a novel class of antineoplastic agents. This modified dipeptidyl boronic acid of only 284 Dalton, is a potent, reversible and specific inhibitor of the 26 S proteasome.

Bortezomib is a cytotoxic anticancer drug and, as with other potentially toxic compounds, caution should be exercised when handling.
If Bortezomib solution contacts the skin, wash the skin immediately and thoroughly with soap, water, and diluted hydrogen peroxide. If Bortezomib solution contacts the mucous membranes, flush thoroughly with water.

In animal studies lethal intravenous doses were associated with decreased blood pressure, increased heart rate, increased cardiac contractility and terminal hypotension.
No cases of overdosage with Bortezomib were reported during clinical trials. Single doses of up to 2 mg/m² have been administered to adults.
In the event of overdosage, patients should be medically monitored and appropriate supportive care given:
- Keep patient warm,
- support blood pressure and
- avoid dehydration.

<u>There is no specific antidot for Bortezomib overdosage.</u>

Bortezomib will be dosed at 1.5 mg/m^2/dose unless adverse events associated with application necessitate dose modification as listed below.

Subcutaneous administration (recommended for all patients unless adverse events like intolerability or unfeasibility (massive edema for example) of the subcutaneous route occur):

Application will be a subcutaneous injection twice weekly on days 1 and 4 of a three-week treatment cycle as described previously [47]. Due to pharmacodynamic considerations and in order to distinguish possible adverse events, <u>Bortezomib should be administered at least 1 hour prior to Rituximab application</u>.
Bortezomib is a sterile lyophilized powder for reconstitution and is supplied in vials containing Bortezomib and mannitol at a 1:10 ratio. Vials should be stored according to the directions provided on the label. Each vial should be reconstituted within 8 hours before dosing with normal (0.9%) saline (e.g.1.4 ml 0.9% saline for a 3.5 mg vial) , so that the reconstituted solution contains 2.5 mg/ml (PLEASE NOTE: The final drug concentration, when reconstituted for SC administration is 2.5 times higher than that for the IV route (1mg/ml) and therefore the volume required is lower when the SC route of administration is used. To avoid administration errors, syringes for SC and IV use should be labeled differently.

Intravenous administration (**only in case of adverse events and after discussion with the LKP / PI**please see chapter 5 Toxicities):

Application will be a 3- to 5-second bolus intravenously twice weekly on days 1 and 4 of a 3-week treatment cycle. Due to pharmacodynamic considerations and in order to distinguish possible adverse events, <u>Bortezomib should be administered at least 1 hour prior to Rituximab application</u>.
Bortezomib is a sterile lyophilized powder for reconstitution and is supplied in vials containing Bortezomib and mannitol at a 1:10 ratio. Vials should be stored according to the directions provided on the label. Each vial should be reconstituted within 8 hours before dosing with normal (0.9%) saline, so that the reconstituted solution contains Bortezomib at a concentration of 1 mg/mL. The reconstituted solution is clear and colorless, with a final pH of 5 to 6. Reconstituted Bortezomib should be administered promptly and in no case more than 8 hours after reconstitution.
Bortezomib will be prepared under aseptic conditions. The amount (in mg) of Bortezomib to be administered will be determined based on BSA. The Bortezomib dose will <u>not</u> be corrected for obese subjects. The dose should be calculated on day 1 of each cycle; the dose administered should remain the same throughout each cycle but should be recalculated at the start of the next cycle.
The appropriate amount of Bortezomib will be administered as an IV push over 3- to 5-seconds followed by a standard saline flush or through a running IV line.

<u>For potential Adverse Effects of Bortezomib please refer to section 1.3.3</u>

3.5.4 DOSE REDUCTION AND TREATMENT DELAY OF BORTEZOMIB

If a Bortezomib dose due to toxicity or another reason is missed, then that dose is skipped and treatment continues with next planned dose.
Observed toxicities, considered by the investigator to be related specifically to Bortezomib are to be managed as follows:

- If leucocytes are < 1.0 x 10^9/l (and/or platelet counts < 25,000 cells/µl), scheduled Bortezomib application will be skipped until recovery to NCI toxicity ≤ 2.
- For any ≥grade 3 non-hematologic toxicity other than neuropathic pain and/or peripheral sensory neuropathy, considered by the investigator to be related to Bortezomib, scheduled Bortezomib application will be skipped until the toxicity returns to Grade 2 or better.
- If a dose of Bortezomib was skipped due to toxicity then reduce the following drug doses as follows:
    - If the patient was receiving 1.5 mg/m^2, reduce the dose to 1.3 mg/m^2.
    - If the patient was receiving 1.3 mg/m^2, reduce the dose to 1.0 mg/m^2.
    - If the patient was receiving 1.0 mg/m^2, reduce the dose to 0.7 mg/m^2
    - If the patient was receiving 0.7 mg/m^2, discontinue study drug, unless patient is responding, in which case this should be discussed with the study coordinators. Dose reductions below 0.7 mg/m^2 should be avoided, but will be considered if patient is having a good response.
- Patients who experience Bortezomib- related neuropathic pain and/or peripheral sensory neuropathy are to be managed as follows:

Neuropathic Pain (NCI CTCAE Grade)		Peripheral Sensory Neuropathy (NCI CTCAE Grade): 0	1	2	3	4
		Normal	Loss of deep tendon reflexes or paresthesia but not interfering with function	Objective sensory loss or paresthesia, interfering with function, but not with ADLs	Sensory loss or paresthesia interfering with ADLs	Disabling
0	None	No action	No action	~25% dose reduction*	Skip; ~50% dose reduction**	Discontinue VELCADE
1	Mild pain not interfering with function	No action	No action	~25% dose reduction*	Skip; ~50% dose reduction**	Discontinue VELCADE
2	Moderate pain: pain or analgesics interfering with function, but not ADLs	~25% dose reduction*	~50% dose reduction	Skip; ~50% dose reduction**	Skip; ~50% dose reduction**	Discontinue VELCADE
3	Severe pain: pain or analgesics severely interfering with ADLs	Skip; ~50% dose reduction**	Skip; ~50% dose reduction**	Skip; ~50% dose reduction**	Discontinue VELCADE	Discontinue VELCADE
4	Disabling	Discontinue VELCADE	Discontinue VELCADE	Discontinue VELCADE	Discontinue VELCADE	Discontinue VELCADE

ADLs = activities of daily living

Key:

Skip: Interrupt Bortezomib (VELCADE™) until the toxicity returns to Grade 1 or better.

*~25% Dose reduction: VELCADE dose reduction from 1.5 to 1.3 mg/m^2/dose, from 1.3 to 1.0 mg/m^2/dose or from 1.0 to 0.7 mg/m^2/dose.

**~50% Dose reduction: VELCADE dose reduction from 1.5 to 1.0 or from 1.3 to 0.7 mg/m^2/dose.

3.5.5 FOLLOW-UP

After completion of salvage therapy, patients will be followed for at least 36 months. As new data indicate a benefit for the patients, if they are given a maintenance or consolidation therapy [46], the decision on administration of maintenance or consolidation therapy is left at the investigator’s discretion. . During that time, regular staging examinations will be performed (see also chapter 4.3.5). If any progress has been detected, patients will be treated by the responsible physician outside of the study according to local guidelines.

3.6 SUPPORTIVE CARE

All medications and procedures and support therapies administered from screening through the end of treatment must be recorded in the source documents and the subject’s CRF as free text.

Patients should receive full supportive care, including hydration and diuresis as needed, transfusions of blood and blood products, antibiotics, anti-emetics, etc. where applicable.

According to a proposed classification of acute emetogenicity of cancer chemotherapy published 1997 by the American Society of Clinical Oncology the emetic potential of Ara-C at doses >1-1.5 g is at level four (i.e. high), indicating a considerable frequency of emesis of up to 90%. Therefore the current antiemetic regimen of choice is

a combination of an effective dose of an 5-HT3 receptor antagonist (e.g. ondansetron, granisetron) and previous dosing of dexamethasone within the R-HAD regimen. Additional dopaminergic antagonist or neurokinin-1-receptor-antagonists may be used as needed.

Use of hematopoietic growth factors is at the choice of each individual investigator. The use of G-CSF might be considered if grade 4 (severe neutropenia) or febrile neutropenia grade III after any cycle of chemotherapy has occurred. G-CSF should be started not before day +5 and continued for 7-10 days, or until the granulocytes have risen to >3x 10^9/l. If Pegfilgrastim is used it should be administered from day +5 on.

The reason(s) for treatment, dosage, and dates of treatment should be recorded on the CRF. Prophylactic antibiotics are not routinely recommended and should be handled according to local standard care.

4 DIAGNOSTIC PROCEDURES

4.1 TABLE OF DIAGNOSTIC TESTS

A schedule of treatment and assessments, that will be carried out during the study is shown.

Visit	*base-line*	*each cycle*[1]	*midterm staging*	*end of treatment evaluation*	*2- year follow-up at 3 month-intervals*	*follow-up thereafter at 6 month-intervals*[15]
Informed consent	X					
Inclusion/exclusion criteria	X					
Lymph node biopsy	X[2]					
MRD[3]	X[4]		x	x		
Medical history [5]	X	X	X	X	X	X
Physical examination [6] (to be documented in patient's file)	X	X	X	X	X	X
Vital signs (to be documented in patient's file)	X	X	X	X	X	X
WHO Performance Status	X (registration)		X (patient's file)	X (patient's file)	X (patient's file)	X (patient's file)
ECG/ Echocardiography	X[7] (patient's file)		X (patient's file)	X (patient's file)		
Radiological tumor assessment (e.g. CT scan)	X[7]		X	X	X	X
Hematologic tests [8]	X	X	X	X	X	X
Serum chemistry [9]	X	X	X	X	X	X
Flow cytometry [10]	X			X	X	X
Bone marrow biopsy	X[11]		X[12]	X[12]	X[12]	X[12]
Serum Test for HIV, hepatitis B and C	X[7]					
Serum pregnancy test [13]	X					
Concomitant medication[14]	X	X	X	X	X	X
Adverse events	X	X	X	X	X	X

1 A deviation of + / - 3 days in the scheduled cycles is considered as per protocol treatment
2 if not performed during 6 months prior to study entry
3 Sampling for minimal residual disease (20 ml EDTA blood, 5 ml EDTA bone marrow, 20 ml heparin blood, 5 ml heparin bone marrow, 4 bone marrow smears (air dried, unfixed for FISH), 10 ml clotted serum sample; details insection 4.3.6
4 only in case of availability of appropriate material
5 including prior antineoplastic therapy, best response, duration of response and adverse events
6 including body weight and height
Baseline: documentation in patient's file / CRF: square meter
During trial: respective CRF pages
7 Only to be performed, if not done within 4 weeks prior to baseline
8 Complete blood counts with differential and platelet count
Please note: although only few data have to be documented on the CRF, all values have to be documented in the patient's file, evaluated (clinical significant / not clinical significant) and signed by a (sub-)investigator

9 Electrolytes, calcium, creatinine, BUN, uric acid, LDH, CRP, glucose, AST(SGOT), ALT (SGPT), alkaline phosphatase, gamma GT, total bilirubin, total protein, albumin. Baseline and End of Treatment evaluation: add IgG, IgM, IgA, ESR, β2 microglobuline, thymidine kinase at baseline and end of treatment evaluation
Please note: the close monitoring of patients during induction treatment is considered as medical standard. Therefore, these values have to be documented as described above in the patient's file but not in the CRF. (Exception: values cause a dose reduction or SAE. In this case, documentation should be done as free text)

10 CD5, CD 19, CD 20 (patient's file)

11 only if not performed during previous 3 month of study entry

12 only to confirm CR if bone marrow was infiltrated initially or if clinically indicated (suspected progression of disease)

13 female patients of child bearing potential only

14 Please use free text for documentation on CRF pages

15 mandatory follow-up period will end at 36 months, however further follow-up is strongly recommended

4.2 SAFETY MEASUREMENTS

Safety evaluation will be done throughout the study, during treatment and follow-up period at each visit:
- Medical history with special emphasis on adverse events.
- Complete physical examination (including vital signs, weight, performance status).
- Laboratory work-up: CBC with differential and platelet count, electrolytes, calcium, creatinine, BUN, uric acid, LDH, CRP, glucose, AST(SGOT), ALT (SGPT), alkaline phosphatase, gamma GT, total bilirubin, total protein, albumin.
- Additional diagnostic studies may be performed according to clinical symptoms based on the judgement of the investigator.

Additional safety evaluation after each therapy cycle:

- The following laboratory work-up must be performed on a weekly base after each cycle: CBC with differential and platelet count, electrolytes, calcium, creatinine, BUN, uric acid, LDH, CRP, glucose, AST (SGOT), ALT (SGPT), alkaline phosphatase, gamma GT, total bilirubin.
- In case of thrombocytopenia grade 4 or neutropenia grade 4 CBC with differential and platelet count should be performed again within four days until recovery from grade 4 toxicity.
- Additional laboratory controls may be performed according to clinical or laboratory symptoms based on the judgement of the investigator

4.3 EVALUATION OF TREATMENT AND RESPONSE

4.3.1 RESPONSE CRITERIA AND TIME SCHEDULE OF REPONSE EVALUATION

Response will be evaluated three weeks after the first two cycles of trial therapy and 4 to 6 weeks after the end of trial therapy. Follow-up staging will be performed every three months during the first two years after end of trial therapy and every six months thereafter. Response is always evaluated in comparison to the status before start of trial therapy. Evaluation of response will be done according to the International Workshop to Standardize Response Criteria for Non-Hodgkin's Lymphoma (see appendix).

4.3.2 BASELINE EVALUATIONS

The following baseline evaluations must be performed and results have to be available prior to start of treatment:
- Written informed consent
- Check of inclusion/exclusion criteria
- Medical history and demographic data
- Prior anti-neoplastic therapy (including best response status and duration of response, residual toxicity)
- Complete physical examination (including vital signs, height and body weight, WHO performance status [refer to appendix], and a neurologic evaluation (preferably done by an experienced neurology consultant)
- ECG, echocardiography
- CT of the cervical region (neck), chest, abdomen and pelvis
  Clinical laboratory examination: CBC with differential and platelet count, electrolytes, calcium, creatinine, BUN, uric acid, LDH, CRP, glucose, AST(SGOT), ALT (SGPT), alkaline phosphatase, gamma GT, total bilirubin, total protein, albumin, IgG, IgM and IgA.
- Erythrocyte sedimentation rate (ESR), β2 microglobuline, thymidine kinase
- Serum test for HIV, HBV and HCV infection
- Serum pregnancy test for female patients of child bearing potential only
- Bone marrow biopsy for cytology, histology and immunophenotyping for lymphoma infiltration (CD19, CD20, CD5 staining) and percentage of involvement if not performed during 3 months prior to study entry
- Immunophenotyping of peripheral blood by flow cytometry with quantitative determination of the total number of B-lymphocytes and circulating lymphoma cells (including CD19, CD20 and CD5 staining)
- Lymph node biopsy if not performed during 6 months prior to study entry
- Only in case of availability of appropriate material: Sampling for minimal residual disease (20 ml EDTA blood, 5 ml EDTA bone marrow, 20 ml heparin blood, 5 ml heparin bone marrow, 4 bone marrow smears (air dried, unfixed for FISH), 10 ml clotted serum sample; details in 4.3.6)
- Additional diagnostic studies should be performed according to clinical symptoms based on the judgement of the investigator.

4.3.3 MIDTERM STAGING PROCEDURES

The following diagnostic procedures have to be performed after 2 treatment cycles, prior to next scheduled treatment:

- Medical history with special emphasis on adverse events.
- Complete physical examination (including vital signs, weight, performance status)
- Laboratory work-up: CBC with differential and platelet count, electrolytes, calcium, creatinine, BUN, uric acid, LDH, CRP, glucose, AST(SGOT), ALT (SGPT), alkaline phosphatase, gamma GT, total bilirubin, total protein, albumin, IgG, IgM and IgA.
- ECG, echocardiography.
- CT of all known lymphoma manifestations
- In case of isolated bone marrow involvement a bone marrow aspiration/ biopsy is mandatory. (Only to confirm CR if bone marrow was infiltrated initially or if clinically indicated (suspected progression of disease))
- Sampling for minimal residual disease (20 ml EDTA blood, 20 ml heparin blood; details in 4.3.6)
- Additional diagnostic studies may be performed according to clinical symptoms based on the judgement of the investigator.

4.3.4 END OF TREATMENT EVALUATION

The following diagnostic procedures have to be performed 4 to 6 weeks after end of treatment (irrespective of number of applied treatment cycles):

- Medical history with special emphasis on adverse events.
- Complete physical examination (including vital signs, weight, performance status)
- Laboratory work-up: CBC with differential and platelet count, electrolytes, calcium, creatinine, BUN, uric acid, LDH, CRP, glucose, AST(SGOT), ALT (SGPT), alkaline phosphatase, gamma GT, total bilirubin, total protein, albumin.
- Erythrocyte sedimentation rate (ESR).
- Immunphenotyping of peripheral blood by flow cytometry with quantitative determination of the total number of B-lymphocytes and circulating lymphoma cells
- ECG, echocardiography.
- CT of neck, chest, abdomen and all other known or suspected lymphoma manifestations
- In case of isolated bone marrow involvement a bone marrow aspiration/ biopsy is mandatory. (Only to confirm CR if bone marrow was infiltrated initially or if clinically indicated (suspected progression of disease))
- Sampling for minimal residual disease (20 ml EDTA blood, 5 ml EDTA bone marrow, 20 ml heparin blood, 5 ml heparin bone marrow, 4 bone marrow smears (air dried, unfixed for FISH); details in 4.3.6)
- Additional diagnostic studies should be performed according to clinical symptoms based on the judgement of the investigator.

4.3.5 FOLLOW-UP EVALUATIONS

The following diagnostic procedures have to be performed at 3 months intervals during the 2-year follow-up and in 6 month intervals thereafter. The mandatory follow-up period will end at 36 months, however in case of continuing response thereafter further follow-up in 6 month intervals is strongly recommended:

- Medical history with special emphasis on adverse events.
- Complete physical examination (including vital signs, weight, performance status)
- Laboratory work-up: CBC with differential and platelet count, electrolytes, calcium, creatinine, BUN, uric acid, LDH, CRP, glucose, AST(SGOT), ALT (SGPT), alkaline phosphatase, gamma GT, total bilirubin, total protein, albumin.
- Erythrocyte sedimentation rate (ESR).
- CT of neck, chest, abdomen (and other known or suspected lymphoma manifestations)
- In case of isolated bone marrow involvement or suspected progress/ relapse a bone marrow aspiration/ biopsy is mandatory.
- Immunphenotyping of peripheral blood by flow cytometry with quantitative determination of the total number of B-lymphocytes and circulating lymphoma cells.
- Sampling for minimal residual disease (at 6 month intervals: 20 ml EDTA blood, 5 ml EDTA bone marrow, 20 ml heparin blood, 5 ml heparin bone marrow, 4 bone marrow smears (air dried, unfixed for FISH), 10 ml clotted serum sample; details in 4.3.6) (if bone marrow aspiration or biopsy was clinically indicated)
- Additional diagnostic studies should be performed according to clinical symptoms based on the judgement of the investigator.

4.3.6 EUROPEAN MCL RESEARCH NETWORK/MINIMAL RESIDUAL DISEASE (MRD)

Based on this previously established *European MCL Intergroup Working Party* and *the European MCL Pathology Panel*, in 2000 a *European MCL Research Network* has been established which focusses especially on the characterization of molecular and biological risk factors in MCL. In addition minimal residual disease will be systematically performed in all study patients. As a prerequisite for molecular assessment of MRD using RQ-PCR peripheral blood and bone marrow has to be sent before start of any treatment to determine the individual patient-specific DNA-sequence of the malignant clone. If no bone marrow material is available at time of inclusion, only peripheral blood may be sent.
Additional FACS and FISH analysis will be performed at every time point of MRD detection. Therefore it is essential to send heparin samples for FACS analysis together with EDTA samples for PCR analysis at every time point of MRD investigation. A 10 ml clotted serum sample will be necessary only at diagnosis.

The results of MRD will be not incorporated into the response evaluation nor influence the management of the patient.

Sample collection
Time points for sample collection for the European MCL Research Network/Biomed II MRD project are:

Prior treatment: for all patients before treatment
20 ml EDTA blood / 5 ml EDTA bone marrow
20 ml heparin blood / 5 ml heparin bone marrow
4 bone marrow smears (air dried, unfixed for FISH)
10 ml clotted serum sample

Midterm staging: after 2 x R-HAD or R-HAD plus Bortezomib
20 ml EDTA blood
20 ml heparin blood

End of treatment: after 4 x R-HAD or R-HAD plus Bortezomib
20 ml EDTA blood / 5 ml EDTA bone marrow
20 ml heparin blood / 5 ml heparin bone marrow
4 bone marrow smears (air dried, unfixed for FISH)

Follow-up (every 3 months during 2 year follow-up, every 6 months thereafter):
at 3-months intervals: 20 ml EDTA blood / 20 ml heparin blood
at 6-months intervals: 20 ml EDTA blood / 5 ml EDTA bone marrow
20 ml heparin blood/ 5 ml heparin bone marrow
4 bone marrow smears (air dried, unfixed for FISH)

Overview of addresses of a selected list of national reference laboratories:

Country	Name and address
France	Prof. Dr. Elizabeth Macintyre, M.D.<br>Laboratoire d'Hematologie Tour Pasteur<br>2ème étage, Porte 14<br>Hôpital Necker-Enfants Malades<br>149, rue de Sevres<br>75743 PARIS CEDEX 15 - France<br>Phone. +33-1-44494947<br>Fax. +33-1-44381745
Germany	Dr. C. Pott<br>Universitätsklinikum Schleswig-Holstein, Campus Kiel<br>Klinik für Innere Medizin II<br>Chemnitzstrasse 33<br>24116 Kiel<br>Phone +49-431-1697-1268<br>Fax. +49-431-1697-1264

Samples with the completed molecular form (Monday to Thursday by express mail) should be sent to the respective national reference labs where samples are centrally collected

5 TOXICITIES

5.1 ADVERSE EVENTS

Special attention is to be paid to the occurrence of adverse events (AE) throughout every stage of the study.
An **adverse event** (AE) is any untoward medical occurrence in a patient administered a pharmaceutical product, which does not necessarily have a causal relationship with the treatment. An adverse event can be any unfavorable and unintended sign (e.g. including an abnormal laboratory finding), symptom, or disease temporally associated with the use of the study drug, whether or not it is considered to be study drug related. This includes any newly occurring event or previous condition that has increased in severity or frequency since the administration of study drug.
Investigators should be familiar with potential adverse events which have been observed in association with the application of Rituximab, high-dose Ara-C, Dexamethasone and Bortezomib as outlined above.

The intravenous route of administration can be selected only in case of intolerability (adverse event) or unfeasibility (e.g. massive edema, cachexia) of the subcutaneous route. In case of local adverse events related to subcutaneous administration of Bortezomib, change of injection site and temporary treatment interruption should be attempted before switching to intravenous administration. Case report form has to be filled appropriately mentioning changes in drug route of administration. The intravenous administration must be approved in writing by the site Principal Investigator. First intravenous application should be made with caution, because the local reaction could be a sign of a drug allergy and subsequent intravenous administrations could potentially cause more serious side effects. Therefore, careful medical observation of the patient is needed to ensure patient's safety.

Adverse events due to the subcutaneous injection of Velcade have to be carefully documented on the respective CRF pages (CRF cycle documentation, remarks –FREETEXT), as this is the only reason for switching back to the intravenous administration. Changes in drug route of administration have to be documented and are only allowed after approval by the Coordinating investigators!

5.2 SERIOUS ADVERSE EVENTS

A serious adverse event (SAE) is defined as any untoward medical occurrence that at any dose:
- results in death, or
- is life-threatening, or

- requires inpatient hospitalization or prolongation of existing hospitalization, or
- results in persistent or significant disability/incapacity, or
- is a congenital anomaly/birth defect, or
- is a suspected transmission of infectious agents by medicinal product, or
- is an important medical event. An important medical event is an event that may not result in death, be life-threatening, or require hospitalization but may be considered an SAE when, based upon appropriate medical judgment, it may jeopardize the patient or subject and may require medical or surgical intervention to prevent one of the outcomes listed in the definitions for SAEs.

All defined Serious Adverse Events (SAEs) occurred after signature of informed consent up to 30 days after the last study drug administration, whether or not ascribed to the IMP, will be reported to the sponsor. A Serious Adverse Event that occurs after this time, if considered related to the study medication, will be reported.

Any serious adverse event that is not listed in 5.3 has to be reported by fax immediately within 24 hours to the sponsor (contact address for SAE reporting: LYSARC, Centre Hospitalier Lyon Sud, Secteur Sainte-Eugénie, pavillon 6D, 165, chemin du Grand Revoyet, 69495 Pierre-Bénite Cedex, France, fax number: +33 3 59 11 01 86, phone number: +33 4 72 66 93 33). All detailed information about the event should be documented on the SAE report form, a referral to the SAE report should be made on the respective CRF page as free text. The following information is required:

- date and time of onset
- duration (date of onset and end)
- peak intensity (according to CTC criteria, **please use CTC AE version 4.03**).
- drug relationship of the AE to the investigational product (for definitions, see below)
- outcome of the adverse event (recovered completely / with residual effects, continuing).
- assessment of the seriousness of the event

The investigator has to classify the drug relationship of an SAE according to the following definitions:

- *None*: The time course between administration of the study drug and occurrence or worsening of the adverse event rules out a causal relationship and/or another cause is confirmed and no indication of involvement of the study drug in the occurrence/ worsening of the adverse event exists.
- *Unlikely*: The time course between administration of the study drug and occurrence or worsening of the adverse event makes a causal relationship unlikely and/or the known effects of the study drug provide no indication of involvement in the occurrence/worsening of the adverse event and another cause adequately explaining the adverse event is known and/or regarding the occurrence/ worsening of the adverse event a plausible causal chain may be deduced from the known effects of the study drug, but another cause is much more probable and/or another cause is confirmed and involvement of the study drug in the occurrence/ worsening of the adverse event is unlikely.
- *Possible*: Regarding the occurrence/worsening of the adverse event a plausible causal chain may be deduced from the pharmacological properties of the study drug, but another cause is just as likely to be involved or although the pharmacological properties of the study drug provide no indication of involvement in the occurrence/worsening of the adverse event, no other cause can be identified.
- *Probable*: The pharmacological properties of the study drug and the course of the adverse event (after rechallenge) and/or specific tests (e.g. positive allergy test, antibodies against study drug/metabolites) suggest involvement of the study drug in the occurrence/worsening of the adverse event, although another cause cannot be ruled out.
- *Definite*: The pharmacological properties of the study drug and the course of the adverse event (after rechallenge) and specific tests (e.g. positive allergy test, antibodies against study drug/metabolites) indicate involvement of the study drug in the occurrence/worsening of the adverse event, and no other causes exists

Monitoring of Adverse Events and Period of Observation

Adverse events, both serious and non-serious, and deaths that occur during the patient’s study participation will be recorded in the source documents. All SAEs should be monitored until they are resolved or are clearly determined to be due to a patient’s stable or chronic condition or intercurrent illness(es).

Procedures for Reporting Drug Exposure During Pregnancy and Birth Events

If a woman becomes pregnant or suspects she is pregnant while participating in this study, she must inform her treating physician immediately and permanently discontinue study drug. The study center must also be contacted immediately by faxing a pregnancy report. The pregnancy must be followed through delivery for SAEs.

If a pregnancy related event is reported in a female partner of a male subject, the investigator should determine whether the female partner is willing to release her medical information to LYSARC Pharmacovigilance and allow the pregnancy related event to be followed-up to completion.

5.3 EXPECTED TREATMENT RELATED SAE

Planned hospital admissions or surgical procedures for an illness or disease which existed before the patient was enrolled in the study are not to be considered SAEs unless the condition deteriorated in an unexpected manner during the study (eg surgery was performed earlier than planned).

Instead the cases mentioned above should be documented on the respective CRF within the standard documentation process.

Alopecia toxicity (any grade) will never be reported as AE.

Signs, symptoms and physical findings indicative of progression of lymphoma are not to be reported as AE and as SAE

6 CAUSES OF DEATH

In the case report form, the cause of death should be documented according to the following listing:

- Mantle cell lymphoma.
- Complication of therapy.
- Intercurrent disease.
- Secondary malignancy.
- Other cause.

7 STATISTICAL METHODS

7.1 STATISTICAL EVALUATION OF THE PRIMARY TRIAL ENDPOINT

The primary endpoint of this trial is the time to treatment failure (TTF) calculated from the date of randomization. Treatment failure is defined as

- progressive disease (PD) or stable disease (SD) following induction therapy or
- relapse or progression after complete or partial remission (CR, CRu, PR) or
- death from any cause,

whichever occurred first. Response to therapy is defined as the staging result after the last cycle of therapy. If no treatment failure has been observed until the time of analysis, TTF is censored at the day of the last follow-up staging.

For ethical reasons, the statistical monitoring of the primary trial endpoint is done with planned interim analyses by means of the truncated sequential probability ratio test as described by Whitehead[(1)]. The logrank test statistics Z and its variance V are calculated according to the formulas (1) and (2) every time when new data with reported events are available.

$$Z = e - \sum_{i=1}^{k} \frac{o_i r_{iC}}{r_i} \quad (1) \qquad V = \sum_{i:r_i>1} \frac{o_i (r_i - o_i) r_{iC} r_{iE}}{r_i^2 (r_i - 1)} \quad (2)$$

with

$t_1,...,t_k$	the distinct, ascendingly ordered failure times
o_i	the number of observed events at t_i
r_i	the number of patients at risk before t_i
r_{iC}	the number of patients at risk before t_i in the R-HAD arm
r_{iE}	the number of patients at risk before t_i in the Bortezomib + R-HAD arm
e	the total number of observed events

If the values of Z and V remain within the boundaries of the continuation region (Figure 1), statistical monitoring is continued. If the values of Z and V leave the continuation region, the statistical test decides against or in favor of the null hypothesis of no difference in TTF between the two study arms, depending on which boundary is crossed. If the values of Z and V cross the upper solid line in Figure 1, the null hypothesis is rejected and Bortezomib + R-HAD considered superior to R-HAD alone. If the values of Z and V cross the lower dotted line, the null hypothesis is rejected and Bortezomib + R-HAD considered inferior to R-HAD alone. If the values of Z and V cross the dashed line, the null hypothesis is accepted. The Christmas tree adjustment for discrete monitoring is performed according to Whitehead [45].

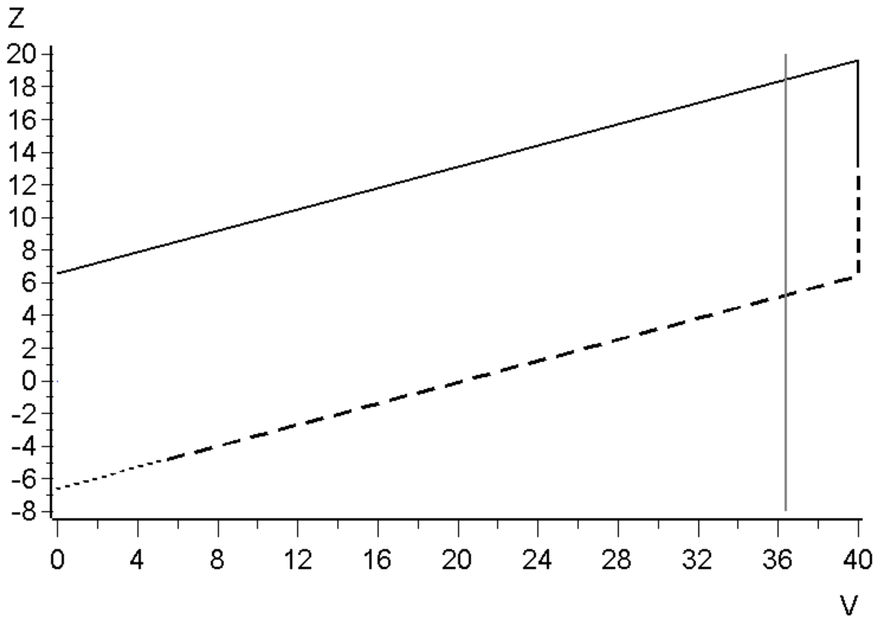

Figure 1: Continuation Region for the Truncated Sequential Probability Ratio Test: intercepts on Z-axis +/- 6.606, slope on upper and lower boundary 0.3259, truncation at V = 40 (160 events), experimental treatment inferior, if lower boundary crossed before V = 5.166.

For the primary analysis patients are evaluated on an intention-to-treat basis. Thus, all patients are analyzed in the treatment arm they have been randomized to, regardless of which treatment they received and whether further protocol violations have occurred. Patients for whom no staging has been performed during induction therapy have to be excluded from analysis. Patients for whom the diagnosis MCL is rejected by the central pathology review are also excluded from analysis.
A secondary analysis on a per-protocol basis is also performed. Patients are evaluable per protocol if they actually received the treatment they were assigned to by randomization and treatment was not stopped prematurely. Thus, patients with progressive (PD) or stable disease (SD) at the end of therapy have to have received at least two cycles and patients with partial (PR, CRu) or complete remission (CR) have to have received the total number of four cycles of therapy.

7.2 NUMBER OF SUBJECTS AND EXPECTED TRIAL DURATION

For the calculation of the required number of events a hazard reduction to 55% by the addition of Bortezomib to R-HAD is considered as a clinically relevant and achievable goal. The maximum number of events is limited to 160 by truncation. With the significance level set to $\alpha = 0.05$ and a desired power of 95% the average number of events needed to obtain a decision of the statistical test is calculated to 87 for a true hazard ratio of 55% (Table 1).

**true hazard ratio**	**55%**	**74%**	**100%**	**182%**
**average number of events**	87	118	76	29
**median number of events**	78	136	66	27
**90% quantile of number of events**	160	160	147	44

Table 1

To obtain the respective sample sizes, the results of the R-FCM arm of the preceding GLSG trial for relapsed or refractory mantle cell lymphoma are used to estimate the distribution of the TTF in the control arm (Figure 2).

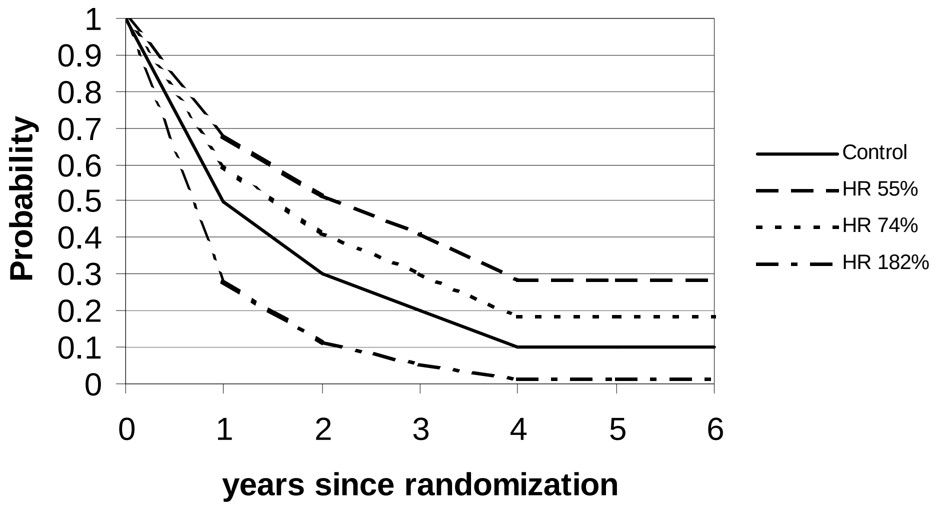

Figure 2: TTF – event free survival curves estimated for control arm and for experimental arm with hazard ratio 55%, 74% and 182% respectively

Finally, to calculate the approximate recruiting times, a recruiting rate of 50 evaluable patients with MCL per year is considered to be realistic. The approximate sample sizes and recruiting times assuming equal group size are shown in Table 2. Thus, in the case of a true hazard ratio of 55% of the combination of Bortezomib and R-HAD as compared to R-HAD alone, an approximate recruiting time of 3.5 years with a total sample size of approximately 175 patients is necessary. The truncation of the statistical test to 160 events yields a maximal sample size of approximately 275 patients and a maximal recruiting time of approximately 5.5 years.

true hazard ratio	55%	74%	100%	182%
expected recruiting time (years)	3.5	4	3	1.5
expected sample size	175	200	150	75
median recruiting time (years)	3.5	4.5	2.5	1.5
median sample size	175	225	125	75
90% quantile of recruiting time (years)	5.5	5	4.5	2
90% quantile of sample size	275	250	225	100

Table 2

The corresponding fixed sample test with no interim analysis would require a total of 146 events (vertical gray line in Figure 1). This number would result in a recruiting time of approximately 5 years and a sample size of approximately 250 patients in the case of a true hazard ratio of 55% (Table 3) .

true hazard ratio	55%	74%	100%	182%
fixed sample recruiting time (years)	5	4.5	4.5	4
fixed sample size	250	225	225	200

Table 3

7.3 STATISTICAL METHODS FOR SECONDARY ANALYSES

The following secondary endpoints are evaluated for interim and final reports.
complete remission rate: the rate of complete remissions (CR) after induction therapy. A CRu is not counted as a complete remission.
overall response (OR) rate: the rate of complete, complete unconfirmed, and partial remissions (CR, CRu, PR) after induction therapy
progression free survival (PFS): time from randomization to first documentation of progression or relapse or death from any cause, whichever occurred first. Patients with no event during follow-up are censored at the day of the last follow-up staging.
progression free survival of responders (PFS of responders) or response duration (RD): time from end of successful (CR, CRu, PR) trial therapy to first documentation of progression or relapse or death from any cause, whichever occurred first. Patients with no event during follow-up are censored at the day of the last follow-up staging.
time to next lymphoma treatment: time from start of trial therapy to the start of next lymphoma treatment outside the protocol. Patients in which no further treatment has been started are censored at the day of the last follow-up staging.

overall survival (OS): time from randomization to death. Patients who were alive at the day of the last contact are censored at that time.
CR-rates and OR-rates are calculated for each treatment arm with the corresponding 95% confidence intervals. Response rates are compared by means of two-sided Fisher‘s exact test. For time to event variables Kaplan-Meier estimates are calculated with 95% confidence intervals as well as median event free survival times. Time to event variables are compared between treatment groups by means of the logrank test.
Secondary efficacy analyses are done both on an intention-to-treat and on a per-protocol basis.
For safety analysis, maximal grades of CTC toxicity during the course of therapy are determined for each patient. The frequencies of CTC grades 0, 1/2, and 3/4 are calculated for each category and compared by means of two-sided Fisher‘s exact test. For safety analysis patients are evaluated „as treated“. Thus, patients have to have received at least one cycle of therapy and are evaluated according to the therapy they actually received.
The primary and secondary endpoints may be additionally analysed in subpopulations according to age, gender, IPI risk group, and number, kind and response to previous lines of therapy.
The significance level is fixed to $\alpha = 0.05$ for all secondary analyses.

7.4 EVALUATION DURING THE TRIAL

Data about recruitment and pooled data for both arms about response to trial therapy, time to treatment failure and overall survival are evaluated every 6 months and reported to the study chairman and co-chairmen. Toxicity is also reported every 6 months in the treatment arms. These data are also reported at the meetings of the study group and may also be reported about the ongoing trial at conferences. Beside toxicity data no other data for the comparison of the arms are disclosed before the decision of the sequential procedure.

7.5 FINAL AND INTERIM REPORTS AND TERMINATION OF THE TRIAL

The first interim report for the comparison of the arms is done when the sequential procedure monitoring the trial has accepted or rejected the null hypothesis. At this point the results are first disclosed at the meeting of the trial steering committee and then to the study group and the randomization is stopped.

8 ADMINISTRATIVE REQUIREMENTS

8.1 GOOD CLINICAL PRACTICE

The study will be conducted in accordance with the International Conference on Harmonisation (ICH) for Good Clinical Practice (GCP) and the appropriate regulatory requirement(s). The investigator will be thoroughly familiar with the appropriate use of the study drug as described in the protocol and Investigator's Brochure. Essential clinical documents will be maintained to demonstrate the validity of the study and the integrity of the data collected. Master files should be established at the beginning of the study, maintained for the duration of the study and retained according to the appropriate regulations.

8.2 ETHICAL CONSIDERATIONS

The study will be conducted in accordance with ethical principles founded in the Declaration of Helsinki. The IRB/IEC will review all appropriate study documentation in order to safeguard the rights, safety and well-being of the patients. The study will only be conducted at sites where IRB/IEC approval has been obtained. The protocol, Summary of product characteristics (SmPC), informed consent, advertisements (if applicable), written information given to the patients (including diary cards), safety updates, annual progress reports, and any revisions to these documents will be submitted to the IRB/IEC by the sponsor or its designee.

8.3 FINANCING AND INSURANCE

The study will be conducted as "Investigator-Initiated Trial". Sponsor is the Klinikum of the Ludwig-Maximilians-University of Munich. Patients will not receive any payments for their participation in the study.
For every patient an insurance has been contracted according to national requirements. For detailed information, please see the patient information or ask the relevant national study coordinator.

8.4. PATIENT INFORMATION AND CONSENT

After the study has been fully explained, written informed consent will be obtained from either the patient or his/her guardian or legal representative prior to study participation. The method of obtaining and documenting the informed consent and the contents of the consent will comply with ICH-GCP and all applicable regulatory requirement(s). The information for the patients will be adopted by the participating groups in the national language of the patient.

8.5. PATIENT CONFIDENTIALITY

In order to maintain patient privacy, all data capture records, study drug accountability records, study reports and communications will identify the patient by initials and the assigned patient number. The investigator will grant monitor(s) and auditor(s) from the sponsor or its designee and regulatory authority(ies) access to the patient's original medical records for verification of data gathered on the data capture records and to audit the data collection process. The patient's confidentiality will be maintained and will not be made publicly available to the extent permitted by the applicable laws and regulations.

For France: The whole of these procedures will be validated by the National Commission of Data processing and Liberté (CNIL). However the authorized representatives of organizations of regulation will be able to possibly consult the medical files in order to confirm the data collected at the time of this study.

For Germany: All data capture records, study drug accountability records, study reports and communications will identify the patient by assigned unique numbers. However if staff of the sponsor is involved in medical consultations for a patient, they will be given access to the identification data of the patient.

8.6 CHANGES TO THE PROTOCOL AND PROTOCOL COMPLIANCE

The investigator will conduct the study in compliance with the protocol given approval/favorable opinion by the IRB/IEC and the appropriate regulatory authority(ies).

Amendments to the final protocol will be initiated by the Sponsor. Prior to their implementation, all applicable approvals / favorable opinions will be obtained.
Deviations from the protocol and immediate implementation of a proposed protocol amendment should not be made, except when the modification is needed to eliminate an immediate hazard(s) to patients.
Any departures from the protocol must be fully documented in the source documents.

8.7 MONITORING

The sponsor will arrange on-site monitoring visits. At these visits, the monitor will compare the data entered into the CRFs with the hospital or clinic records (source documents). The nature and location of all source documents will be identified to ensure that all sources of original data required to complete the CRF are known to the sponsor and investigational staff and are accessible for verification by the sponsor site contact. At a minimum, source documentation must be available to substantiate: subject identification, eligibility and participation; proper informed consent procedures; dates of visits; adherence to protocol procedures; records of safety and efficacy parameters; adequate reporting and follow-up of adverse events; administration of concomitant medication; drug receipt/dispensing/return records; study drug administration information; date of subject completion, discontinuation from treatment, or withdrawal from the study, and the reason if appropriate. Specific items required as source documents will be reviewed with the investigator before the study. Direct access to source documentation (medical records) must be allowed for the purpose of verifying that the data recorded in the CRF are consistent with the original source data. The sponsor expects that, during monitoring visits, the relevant investigational staff will be available, the source documentation will be available, and a suitable environment will be provided for review of study-related documents.

8.8 ON SITE AUDITS

Regulatory authorities, the IEC/IRB and/or the sponsor or its designee's clinical quality assurance group may request access to all source documents, data capture records, and other study documentation for on-site audit or inspection. Direct access to these documents must be guaranteed by the investigator, who must provide support at all times for these activities. The patient's confidentiality will be maintained and will not be made publicly available to the extent permitted by the applicable laws and regulations.

8.9 DRUG ACCOUNTABILITY

Accountability for the study drug at all study sites is the responsibility of the principal investigator. The investigator will ensure that the study drug is used only in accordance with this protocol. Drug accountability records indicating the drug's delivery date to the site, inventory at the site, use by each patient, and disposal of the drug will be maintained by the clinical site. Accountability records will include dates, quantities, lot numbers, expiration dates (if applicable), and patient numbers.
All used, unused or expired study drug will be disposed of at the study site and documented. All material containing VELCADE will be treated and disposed of as hazardous waste in accordance with governing regulations.

8.10 PREMATURE CLOSURE OF THE STUDY

This study may be prematurely terminated, if in the opinion of the investigator or the sponsor or its designee, there is sufficient reasonable cause. Written notification documenting the reason for study termination will be provided to the investigator or the sponsor or its designee by the terminating party.
Circumstances that may warrant termination include, but are not limited to:

- Determination of unexpected, significant, or unacceptable risk to patients
- Failure to enter patients at an acceptable rate
- Insufficient adherence to protocol requirements
- Insufficient complete and/or evaluable data
- Plans to modify, suspend or discontinue the development of the study drug

Should the study be closed prematurely, all study materials must be returned to the sponsor or its designee.

8.11 END OF STUDY

The end of study is defined as the date of the last visit of the last patient undergoing the study. Within 90 days of the end of the study the sponsor will notify the competent authorities and the ethics committees in all Member States where the study is being carried out that the study has ended. If the study is terminated early the sponsor will notify the competent authorities and ethics committees in all Member States within 15 days and explain the reasons for premature termination.
Within one year of the end of the study a summary of the clinical trial report will be submitted to the competent authorities and ethics committees in all Member States involved.

8.12 RECORD RETENTION

The investigator will maintain all study records according to ICH-GCP and applicable regulatory requirement(s).

8.13 PUBLICATION POLICY

All publication procedures must be in line with the protocol and contract. The final publication of the trial results will be written by the Study Coordinator(s) on the basis of the statistical analysis performed at the Data in Munich. A draft manuscript will be submitted to the Data Center and all co-authors for review. After revision by the Data Centre and the other co-authors, the manuscript will be sent to a peer reviewed scientific journal. Authors of the manuscript will include the study coordinator(s), the lead investigators of the major groups, investigators of most recruiting institutions (by order of inclusion), the statistician, and others who have made significant scientific contributions. Interim publications or presentations of the study may include demographic data, overall results and prognostic factor analyses, but no comparisons between randomized treatment arms may be made publicly available before the recruitment is discontinued. Any publication, abstract or presentation based on patients included in this study must be approved by the study coordinator(s). This is applicable to any individual patient registered in the trial, or any subgroup of the trial patients. Such a publication cannot include an analysis of any of the study end-points unless the final results of the trial have already been published.

8.14 NATIONAL REQUIREMENTS

The study will be conducted at different national sites in accordance with the national law and national regulatory requirements. All documents necessary for the initiation of the study at a national site will be conducted in a separate attachment to this protocol.

9 REFERENCES

1. Jaffe, E.S., Harris, N.L.Stein, H., *Tumours of the Haemopoitic and Lymphoid Tissues*. World Health Organization Classification of Tumours. 2001, Lyon: IARC Press.
2. Rimokh, R., Berger, F., Delsol, G., et al., *Rearrangement and overexpression of the BCL-1/PRAD-1 gene in intermediate lymphocytic lymphomas and in t(11q13)-bearing leukemias.* Blood, 1993. **81**(11): p. 3063-7.
3. Hiddemann, W., Unterhalt, M., Herrmann, R., et al., *Mantle-cell lymphomas have more widespread disease and a slower response to chemotherapy compared with follicle-center lymphomas: results of a prospective comparative analysis of the German Low-Grade Lymphoma Study Group.* J Clin Oncol, 1998. **16**(5): p. 1922-30.
4. Dreyling, M. Hiddemann, W., *Prognostic factors in mantle cell lymphoma: Clinical characteristics, pathology and cell proliferation.* Journal of Clinical Oncology, 1999. **18**: p. 3a.
5. Dreyling, M., Lenz, G., Hoster, E., et al., *Early consolidation by myeloablative radiochemotherapy followed by autologous stem cell transplantation in first remission significantly prolongs progression-free survival in mantle-cell lymphoma: results of a prospective randomized trial of the European MCL Network.* Blood, 2005. **105**(7): p. 2677-84.
6. Lenz, G., Dreyling, M., Schiegnitz, E., et al., *Moderate increase of secondary hematologic malignancies after myeloablative radiochemotherapy and autologous stem-cell transplantation in patients with indolent lymphoma: results of a prospective randomized trial of the German Low Grade Lymphoma Study Group.* J Clin Oncol, 2004. **22**(24): p. 4926-33.
7. Velasquez, W.S., Cabanillas, F., Salvador, P., et al., *Effective salvage therapy for lymphoma with cisplatin in combination with high-dose Ara-C and dexamethasone (DHAP).* Blood, 1988. **71**(1): p. 117-22.
8. Press, O.W., Livingston, R., Mortimer, J., et al., *Treatment of relapsed non-Hodgkin's lymphomas with dexamethasone, high-dose cytarabine, and cisplatin before marrow transplantation.* J Clin Oncol, 1991. **9**(3): p. 423-31.
9. Khouri, I.F., Romaguera, J., Kantarjian, H., et al., *Hyper-CVAD and high-dose methotrexate/cytarabine followed by stem-cell transplantation: an active regimen for aggressive mantle-cell lymphoma.* J Clin Oncol, 1998. **16**(12): p. 3803-9.
10. Lefrere, F., Delmer, A., Suzan, F., et al., *Sequential chemotherapy by CHOP and DHAP regimens followed by high-dose therapy with stem cell transplantation induces a high rate of complete response and improves event-free survival in mantle cell lymphoma: a prospective study.* Leukemia, 2002. **16**(4): p. 587-93.
11. Mey, U., Strehl, J., Orlopp, K., et al., *Dexamethason, Hoch-Dosis Cytarabin und Cisplatin (DHAP) in Kombination mit Rituximab als Salvage-Chemotherapie bei Patienten mit rezidivierten oder primär refraktären aggressiven Non-Hodgkin-Lymphomen - Ergebnisse einer multizentrischen Phase II Studie.* Onkologie, 2004. **27**(Suppl 3): p. 0112 [abstract].
12. Geisler, C.H., Elonen, E., Kolstad, A., et al. *Nordic Mantle Cell Lymphoma (MCL) Project: Prolonged Follow-Up of 86 Patients Treated with BEAM/BEAC + PBSCT Confirms That Addition of High-Dose Ara-C and Rituximab to CHOP Induction + In-Vivo Purging with Rituximab Increases Clinical and Molecular Response Rates, PCR-Neg. Grafts, Failure-Free, Relapse-Free and Overall Survival.* in *ASH*. 2004.
13. Campo, E., Raffeld, M.Jaffe, E.S., *Mantle-cell lymphoma.* Semin Hematol, 1999. **36**(2): p. 115-27.
14. Foran, J.M., Cunningham, D., Coiffier, B., et al., *Treatment of mantle-cell lymphoma with Rituximab (chimeric monoclonal anti-CD20 antibody): analysis of factors associated with response.* Ann Oncol, 2000. **11**(Suppl 1): p. 117-21.
15. Coiffier, B., Haioun, C., Ketterer, N., et al., *Rituximab (anti-CD20 monoclonal antibody) for the treatment of patients with relapsing or refractory aggressive lymphoma: a multicenter phase II study.* Blood, 1998. **92**(6): p. 1927-32.
16. Ghielmini, M., Hsu Schmitz, S.-F., Cogliatti, S., et al., *Effect of standard or prolonged treatment with single agent Rituximab in patients with mantle cell lymphoma. A randomized trial of the SAKK.* The Hematology Journal, 2003. **4**(Supplement 2).
17. Lenz, G., Dreyling, M., Hoster, E., et al., *Immunochemotherapy with rituximab and cyclophosphamide, doxorubicin, vincristine, and prednisone significantly improves response and time to treatment failure, but not long-term outcome in patients with previously untreated mantle cell lymphoma: results of a prospective randomized trial of the German Low Grade Lymphoma Study Group (GLSG).* J Clin Oncol, 2005. **23**(9): p. 1984-92.
18. Forstpointner, R., Dreyling, M., Repp, R., et al., *The addition of rituximab to a combination of fludarabine, cyclophosphamide, mitoxantrone (FCM) significantly increases the response rate and prolongs survival as compared to FCM alone in patients with relapsed and refractory follicular and mantle cell lymphomas - results of a prospective randomized study of the German low grade lymphoma study group (GLSG).* Blood, 2004. **29**: p. 29.
19. Romaguera, J., Cabanillas, F.Dang, N., *Mantle cell lymphoma (MCL) - Update on results after R-HCVAD without stem cell Transplantat (SCT).* Ann Oncol, 2002. **13**: p. 8 [abstract].
20. Khouri, I.F., Lee, M.S., Saliba, R.M., et al., *Nonablative allogeneic stem-cell transplantation for advanced/recurrent mantle-cell lymphoma.* J Clin Oncol, 2003. **21**(23): p. 4407-12.
21. Ciechanover, A., *The ubiquitin proteolytic system and pathogenesis of human diseases: a novel platform for mechanism-based drug targeting.* Biochem Soc Trans, 2003. **31**(2): p. 474-81.

22. Ciechanover, A. Schwartz, A.L., *The ubiquitin-proteasome pathway: the complexity and myriad functions of proteins death.* Proc Natl Acad Sci U S A, 1998. **95**(6): p. 2727-30.
23. Ciechanover, A., *The ubiquitin-proteasome pathway: on protein death and cell life.* Embo J, 1998. **17**(24): p. 7151-60.
24. Baumeister, W., Walz, J., Zuhl, F., et al., *The proteasome: paradigm of a self-compartmentalizing protease.* Cell, 1998. **92**(3): p. 367-80.
25. Glickman, M.H. Ciechanover, A., *The ubiquitin-proteasome proteolytic pathway: destruction for the sake of construction.* Physiol Rev, 2002. **82**(2): p. 373-428.
26. Markopoulos, M., *PS-341 (bortezomib) For Injection.* Millennium Pharmaceuticals, Inc. Investigator Brochure, 2003.
27. Papandreou, C.N., Daliani, D.D., Nix, D., et al., *Phase I trial of the proteasome inhibitor bortezomib in patients with advanced solid tumors with observations in androgen-independent prostate cancer.* J Clin Oncol, 2004. **22**(11): p. 2108-21.
28. Orlowski, R.Z., Stinchcombe, T.E., Mitchell, B.S., et al., *Phase I trial of the proteasome inhibitor PS-341 in patients with refractory hematologic malignancies.* J Clin Oncol, 2002. **20**(22): p. 4420-7.
29. Aghajanian, C., Soignet, S., Dizon, D.S., et al., *A phase I trial of the novel proteasome inhibitor PS341 in advanced solid tumor malignancies.* Clin Cancer Res, 2002. **8**(8): p. 2505-11.
30. O'Connor, O.A., *The emerging role of bortezomib in the treatment of indolent non-Hodgkin's and mantle cell lymphomas.* Curr Treat Options Oncol, 2004. **5**(4): p. 269-81.
31. Goy, A. Gilles, F., *Update on the proteasome inhibitor bortezomib in hematologic malignancies.* Clin Lymphoma, 2004. **4**(4): p. 230-7.
32. O'Connor, O.A., Wright, J., Moskowitz, C., et al., *Phase II clinical experience with the novel proteasome inhibitor bortezomib in patients with indolent non-Hodgkin's lymphoma and mantle cell lymphoma.* J Clin Oncol, 2005. **23**(4): p. 676-84. Epub 2004 Dec 21.
33. Goy, A., Younes, A., McLaughlin, P., et al., *Phase II study of proteasome inhibitor bortezomib in relapsed or refractory B-cell non-Hodgkin's lymphoma.* J Clin Oncol, 2005. **23**(4): p. 667-75.
34. Assouline, S. *A phase II study of Bortezomib in patients with mantle cell lymphoma*. in *ASH 2003*. 2003.
35. Orlowski, R.Z., Voorhees, P.M., Garcia, R.A., et al., *Phase 1 trial of the proteasome inhibitor bortezomib and pegylated liposomal doxorubicin in patients with advanced hematologic malignancies.* Blood, 2005. **105**(8): p. 3058-3065.
36. Dunleavy, K., Janik, J.Wilson, W. *Phase I/II Study of Bortezomib with Dose-Adjusted EPOCH Chemotherapy in Relapsed or Refractory Aggressive B-Cell Lymphoma*. in *ASH*. 2004.
37. Weigert, O., Rieken, M., Zimmermann, Y., et al., *Inhibition of proteasome activity in mantle cell lymphoma is associated with early alterations of cell cycle regulators and enhances chemotherapy induced apoptosis.* Ann Oncol, 2005. **16**(Suppl. 5): p. v81 [abstract #149].
38. Weigert, O., Rieken, M., Zimmermann, Y., et al., *The proteasome inhibitor bortezomib induces rapid alterations of cell cycle regulators and augments sensitivity to cytostatic drugs in mantle cell lymphoma.* Journal of Clinical Oncology, 2005. **23**(16S): p. 578s [abstract#6609].
39. Leonard, J., Furman, R., Cheung, Y.-K., et al., *Phase I/II Trial of Bortezomib + CHOP-Rituximab in Diffuse Large B Cell (DLBCL) and Mantle Cell Lymphoma (MCL): Phase I Results.* Blood, 2005. **106**(147a).
40. Mounier, N., Ribrag, V., C., H., et al., *Efficacy and toxicity of two schedules of R-CHOP plus bortezomib in front-line B lymphoma patients: A randomized phase II trial from the Groupe d'Etude des Lymphomes de l'Adulte (GELA).* Journal of Clinical Oncology, 2007. **25**(abstract #8010).
41. Gerecitano, J., Portlock, C., Noy, A., et al., *The Schedule Dependent Combination of Bortezomib (Bor) with Rituximab (R), Cyclophosphamide (C) and Prednisone (P) Produces Minimal Toxicity, Even at Relatively High Doses of Proteasome Inhibitor, in Patients with Relapsed/Refractory Indolent B-Cell Lymphomproliferative Disorders.* Blood, 2006. **108**(abstract #2759).
42. Weigert, O., Pastore, A., Rieken, M., et al., *Sequence-dependent synergy of the proteasome inhibitor bortezomib and cytarabine in mantle cell lymphoma.* Leukemia, 2007. **21**: p. 524-528.
43. Weigert, O., Weidmann, E., Mueck, R., et al., *High Dose Cytarabine Salvage Regimen Combined with Bortezomib Is Feasible and Highly Effective in Relapsed Mantle Cell Lymphoma.* Blood, 2006. **108**((suppl. 11), # 2449, 693a).
44. Dreyling, M., Hoster, E., Hermine, O., et al., *European MCL Network: An Update on Current First Line Trials.* Blood, 2007. **110**(110 Issue 11 # 388).
45. Whitehead, J., *The Design and Analysis of Sequential Clinical Trials*. 2nd edition ed. 1997: Whiley.
46. Forstpointner, R. et al., *Maintenance therapy with rituximab leads to a significant prolongation of response duration after salvage therapy with a combination of rituximab, fludarabine, cyclophosphamide, and mitoxantrone (R-FCM) in patients with recurring and refractory follicular and mantle cell lymphomas: results of a prospective randomized study of the German Low Grade Lymphoma Study Group (GLSG)*. Blood 2006. **108**(13): p. 4003 - 4008.
47. Simpson, D., et al., *Weekly Subcutaneous Bortezomib Is Well Tolerated and Effective As Initial Therapy Of Symptomatic Myeloma*. ASH 2013. Oral and Poster Abstracts, Session 653. Myeloma: Therapy, excluding Transplantation: Poster II. Sunday, December 8, 2013, 6:30 PM - 8:30 PM.

10 ABBREVIATIONS

AE	Adverse event
ALAT (SGPT)	Alanine aminotransferase
ASAT (SGOT)	Aspartate aminotransferase
ASCT	Autologous stem cell transplantation
BUN	Blood urea nitrogen
CBC	Complete blood count
CNS	Central nervous system
CR	Complete response
CRF	Case report form
Cru	Complete response/unconfirmed
CTC	Common toxicity criteria
ECG	Electrocardiogram
FL	Follicular lymphoma
GCP	Good Clinical Practice
G-CSF	Filgastrim
GMP	Good Manufacturing Process
HAMA	Human anti-murine antibodies
Hb	Hemoglobin
HIV	Human immunodeficiency virus
IV	Intravenous
MCL	Mantel cell lymphoma
MM	Millimole
PBS	Phosphate buffered saline
PBSCT	Peripheral blood stem cell transplantation
PD	Progressive disease
PFS	Progression-free-survival
PPD	Product of two largest perpendicular diameters
PR	Partial response
PS	Performance status
SAE	Serious adverse event
SC	Stem cell
SPD	Sum of the products of the greatest diameters
ULN	Upper limits of normal

11 APPENDIX

11.1 WHO PERFORMANCE CRITERIA

**Definition**	**Grade**
The patient is able to carry out all normal activities without restriction	0
The patient is restricted in physically strenuous activity but able to carry out light work, patient is ambulatory	1
Patient is ambulatory and capable of all self-care but unable to carry out any work, patient is out of bed more than 50% of waking hours	2
Patient is capable of only limited self-care and confined to bed or chair more than 50% of waking hours	3
Patient is completely disabled; cannot carry out any self-care and is totally confined to bed or chair	4

11.2 ECOG PERFORMANCE CRITERIA

**Definition**	**Grade**
Fully active, able to carry on all pre-disease performance without restriction	0
Restricted in physically strenuous activity but ambulatory and able to carry out work of a light or sedentary nature, e.g., light house work, office work	1
Ambulatory and capable of all selfcare but unable to carry out any work activities. Up and about more than 50% of waking hours	2
Capable of only limited selfcare, confined to bed or chair more than 50% of waking hours	3
Completely disabled. Cannot carry on any selfcare. Totally confined to bed or chair	4
Dead	5

11.3 CATEGORIES OF STAGING (ACCORDING TO ANN ARBOR)

Stage I

I: Involvement of a single lymph node region

IE: Localized involvement of a single extralymphatic organ or site

Stage II

II: Involvement of 2 or more lymph node regions on the same side of the diaphragm

IIE: Localized involvement of a single associated extralymphatic organ or site and its regional lymph nodes with or without other lymph node regions on the same side of the diaphragm

Stage III

III: Involvement of lymph node regions on both sides of the diaphragm

IIIE: accompanied by: localized involvement of an extralymphatic organ or site

IIIS: involvement of the spleen

IIIS+E: both (IIIS+IIIE).

Stage IV

IV: disseminated (multifocal) involvement of 1 or more extralymphatic sites with or without
associated lymph node involvement or isolated extralymphatic organ involvement with distant (nonregional) nodal involvement.

IV E: extranodal lymphoid malignancies arise in tissues separate from, but near, the major lymphatic aggregates.

In the absence or presence of fever (> 38°C, not otherwise explained), night sweats (recurrent, drenching) and/or unexplained loss of 10 percent or more of body weight in the six months preceding admission are to be denoted in all cases by the suffix letters A or B, respectively.

11.4 RESPONSE CRITERIA

Evaluation of response will be done according to the International Workshop to Standardize Response Criteria for Non-Hodgkin’s Lymphoma.
In the future, as additional radiographic, laboratory, and functional studies become more widely available and clearly demonstrate predictive value, they may be recommended as well.

CR requires the following:

Complete disappearance of all detectable clinical and radiographic evidence of disease and disappearance of all disease-related symptoms if present before therapy, and normalisation of those biochemical abnormalities (e.g. lactate dehydrogenase (LDH) definitely assignable to NHL.
All lymph nodes and nodal masses must have regressed to normal size (<1.5 cm in their greatest transverse diameter for nodes > 1.5 cm before therapy). Previously involved nodes that were 1.1 to 1.5 cm in their greatest transverse diameter before treatment must have decreased to 1 cm in their greatest transverse diameter after treatment, or by more than 75% in the sum of the products of the greatest diameters (SPD).
The spleen, if considered to be enlarged before therapy on the basis of a CT scan, must have regressed in size and must not be palpable on physical examination. However, no normal size can be specified because of the difficulties in accurately evaluating splenic and hepatic size. For instance, spleens thought to be of normal size may contain lymphoma, whereas an enlarged spleen may not necessarily reflect the presence of lymphoma but variations in anatomy, blood volume, the use of hematopoietic growth factors, or other causes. Any macroscopic nodules in any organs detectable on imaging techniques should no longer be present. Similarly, other organs considered to be enlarged before therapy due to involvement by lymphoma, such as liver and kidneys, must have decreased in size.
If the bone marrow was involved by lymphoma before treatment, the infiltrate must be cleared on repeat bone marrow aspirate and biopsy of the same site. The sample on which this determination is made must be adequate (>20 mm biopsy core). Flow cytometric, molecular, or cytogenetic studies arenot considered part of routine assessment to document persistent disease at the present time.

CR/unconfirmed (CR_u) includes those patients who achieve a complete disappearance of all clinical symptoms or organ involvement, but with one or more of the following features:
A residual lymph node mass greater than 1.5 cm greatest transverse diameter that has regressed by more than 75% in the SPD. Individual nodes that were previously confluent must have regressed by more than 75% in their SPD compared with the size of the original mass.
Indeterminate bone marrow (increased number or size of aggregates without cytological or architectural atypia)

PR requires the following:
50% decrease in SPD of the six largest dominant nodes or nodal masses. These nodes or masses should be selected according to the following features:
- they should be clearly measurable in at least two perpendicular dimensions,
- they should be from as completed disparate regions of the body as possible, and
- they should include mediastinal and retroperitoneal areas of disease whenever these sites are involved.
- No increase in the size of the other nodes, liver, or spleen.
- Splenic and hepatic nodules must regress by at least 50% in the SPD.
- With the exception of splenic and hepatic nodules, involvement of other organs is considered assessable and not measurable disease.
- Bone marrow assessment is irrelevant for determination of a PR because it is assessable and not measurable disease; however, if positive, the cell type should be specified in the report and preferably confirmed by immunohistochemistry.
- No new sites of disease.

Stable disease/No change is defined as less than a PR (see above) but is not progressive disease (see below).

Relapsed disease (after CR, CR_u) requires the following:
- Appearance of any new lesion or increase by > 50% in the size of previously involved sites.
- >50% increase in greatest diameter of any previously identified node greater than 1 cm in its short axis or in the SPD of more than one node.

Progressive disease (after PR, non-responders) requires the following:
- >50% increase from nadir in the SPD of any previously identified abnormal node for PRs or non-responders.
- Appearance of any new lesion during or at the end of therapy

Response is currently assessed on the basis of clinical, radiologic, and pathologic (ie, bone marrow) criteria. PET scanning is not accepted as the sole instrument for response measurements.
CT scans remain the standard for evaluation of nodal disease. Thoracic, abdominal, and pelvic CT scans are recommended even if those areas were not initially involved because of the unpredictable pattern of recurrence in NHL. Radiologic investigations should be performed no later than 4 weeks after the end of treatment to assess response. A bone marrow aspirate and biopsy should only be performed to confirm a CR if they were initially positive or if it is clinically indicated by new abnormalities in the peripheral blood counts or blood smear.

Table 1. Response Criteria for Non-Hodgkin's Lymphoma

**Response category**	**Physical examination**	**Lymph nodes**	**Lymph node masses**	**Bone marrow**	
CR	Normal	Normal	Normal	Normal	
CR_u	Normal	Normal	Normal	Indeterminate	
	Normal	Normal	> 75% decrease	Normal/ indeterminate	
PR	Normal	Normal	Normal	Positive	
	Normal	>50% decrease	>50% decrease	Irrelevant	>
	Decrease in liver/spleen	>50% decrease	≥50% decrease	Irrelevant	
Relapse/ progression	Enlarg.liver/spleen/new sites	New/increased	New/increased	Reappearance	

In case of relapse after initial complete remission the following information will be registered:
Date of relapse
Site of relapse
Whether there is histological or cytological confirmation of the relapse.
